# cAMP controls the balance between dormancy and activation of primordial follicles in mouse ovaries

**DOI:** 10.1093/pnasnexus/pgad055

**Published:** 2023-02-21

**Authors:** Wenying Zheng, Tuo Zhang, Ting Zhao, Zijian Zhu, Shaogang Qin, Hao Yan, Meina He, Bo Zhou, Guoliang Xia, Hua Zhang, Chao Wang

**Affiliations:** State Key Laboratory of Agrobiotechnology, College of Biological Sciences, China Agricultural University, Beijing 100193, China; Guizhou Provincial Key Laboratory of Pathogenesis and Drug Research on Common Chronic Diseases, Department of Physiology, College of Basic Medicine, Guizhou Medical University, Guiyang, Guizhou Province 550025, China; State Key Laboratory of Agrobiotechnology, College of Biological Sciences, China Agricultural University, Beijing 100193, China; State Key Laboratory of Agrobiotechnology, College of Biological Sciences, China Agricultural University, Beijing 100193, China; State Key Laboratory of Agrobiotechnology, College of Biological Sciences, China Agricultural University, Beijing 100193, China; State Key Laboratory of Agrobiotechnology, College of Biological Sciences, China Agricultural University, Beijing 100193, China; Guizhou Provincial Key Laboratory of Pathogenesis and Drug Research on Common Chronic Diseases, Department of Physiology, College of Basic Medicine, Guizhou Medical University, Guiyang, Guizhou Province 550025, China; State Key Laboratory of Agrobiotechnology, College of Biological Sciences, China Agricultural University, Beijing 100193, China; State Key Laboratory of Agrobiotechnology, College of Biological Sciences, China Agricultural University, Beijing 100193, China; Key Laboratory of Ministry of Education for Conservation and Utilization of Special Biological Resources in the Western China, College of Life Science, Ningxia University, Yinchuan 750021, China; State Key Laboratory of Agrobiotechnology, College of Biological Sciences, China Agricultural University, Beijing 100193, China; State Key Laboratory of Agrobiotechnology, College of Biological Sciences, China Agricultural University, Beijing 100193, China

**Keywords:** primordial follicle, dormancy, activation, cAMP, oocyte

## Abstract

In mammalian ovaries, the balance between dormancy and activation of primordial follicles determines the female fecundity and endocrine homeostasis. Recently, several functional molecules and pathways have been reported to be involved in the activation of primordial follicles. However, the homeostasis regulatory mechanisms of primordial follicle activation are still scant. Our previous study has proved that a relatively higher concentration of cyclic AMP (cAMP) is required for primordial follicle formation. Here, we identified that cAMP also plays a vital role in the balance between dormancy and activation of primordial follicles. Our results showed that the concentration of cAMP remained stable in neonatal mouse ovaries, which is due to ADCY3, the synthetase of cAMP, and PDE3A, the hydrolytic enzyme of cAMP, were synchronously increased during the activation of primordial follicles in mouse ovaries. Once the concentration of cAMP in neonatal ovaries was either elevated or reduced in vitro, the activation of primordial follicles was either accelerated or decelerated accordingly. In addition, a higher concentration of cAMP in the ovaries of puberty mice improved primordial follicle activation in vivo. Finally, cAMP promoted primordial follicle activation via canonical mTORC1–PI3K signaling cascades and PKA signaling. In conclusion, our findings reveal that the concentration of cAMP acts as a key regulator in balancing the dormancy and activation of primordial follicles in the mouse ovary.

**Significance Statement**

In mammal, primordial follicles need to maintain the balance between dormancy and activation for sustaining physiological reproductive lifespan. However, the cellular and molecular mechanisms of this are not well elucidated. In this study, we report that cAMP, a well-characterized second messenger, contributes to the homeostasis of primordial follicle activation. Changing the concentration of cAMP either in the ovaries of neonatal mice in vitro or in the ovary cortex of puberty mice in vivo result in the activation of primordial follicles is elevated or inhibited, accordingly. These findings shed new lights on the physiology of sustaining female reproduction.

## Introduction

The ovarian follicle is the basic functional unit for female reproduction. Most follicles in the ovary are dormant primordial follicles, which are the initial stage of follicular development and are nonrenewable sources of reproduction (1, 2). In adults, only a limited portion of primordial follicles are gradually recruited into the growing pool through the activation of primordial follicles (3), which is beneficial for the production of mature oocytes to support the long-term reproductive lifespan of female mammals (4). Once the homeostasis of primordial follicle activation is disrupted, it will inevitably lead to a series of problems such as abnormal follicular development and ovulation disorders (5). Thus, uncovering the mechanism of the balance between dormancy and activation of primordial follicles is vital for improving the longevity and health of female reproduction.

Studies, including ours, have shown that several intracellular/extracellular factors are involved in regulating the activation of primordial follicles. Several signaling pathways have been revealed to activation or maintenance the primordial follicles, such as mechanistic target of rapamycin complex 1 (mTORC1) in granulosa cells and phosphoinositide 3-kinase (PI3K) in oocytes, respectively (6–8). Depending on these signaling pathways, lim homeobox 8 (LHX8), cell division cycle 42 (CDC42), sirtuin 1 (SIRT1), as well as E-cadherin/N-cadherin (E-cad/N-cad) and histone deacetylase 6 (HDAC6) are pivotal for regulating primordial follicle activation (9–13). In addition, nutritional and paracrine factors in the ovarian microenvironment are involved in this process (14, 15). Despite these, the understanding of the mechanisms that balance the activation and dormancy of primordial follicles is still in its infancy.

As a well-characterized second messenger, cyclic AMP (cAMP) responds to extracellular signals by changing the concentration within the cell, which in turn regulates intracellular signal transduction pathways, and thus alters the physiological processes such as cell proliferation, migration, and apoptosis (16, 17). Importantly, the meiosis process of oocytes during follicular development is closely linked to the concentration of cAMP as well. A higher cAMP concentration in the cytoplasm of oocytes of growing follicles is the key point to the arrest of meiosis (18–20). Only when the concentration of the cAMP in oocyte is reduced in the antral follicles, the oocyte resumes meiosis and matures (21). Recently, we found that the concentration of cAMP in fetal ovaries increases as oocytes enter the early meiosis, which deeply affects the establishment of the primordial follicle pool in mice (22). However, the role of cAMP during primordial follicle activation is unknown to date.

This study aims to uncover the functional role of cAMP in the primordial follicle activation. We found that an elevation of the concentration of cAMP either in the ovaries of neonatal mouse in vitro or in the ovary cortex of puberty mouse in vivo increased the activation of primordial follicles. The effect of cAMP in primordial follicle activation is implemented via the activity of mTORC1/PI3K and PKA signaling pathways. Hence, the concentration of cAMP is pivotal for balancing the dormancy and activation of primordial follicles in the mouse ovary.

## Results

### The concentration of cAMP is stable after the formation of primordial follicles in neonatal ovaries

**Fig. 1.**
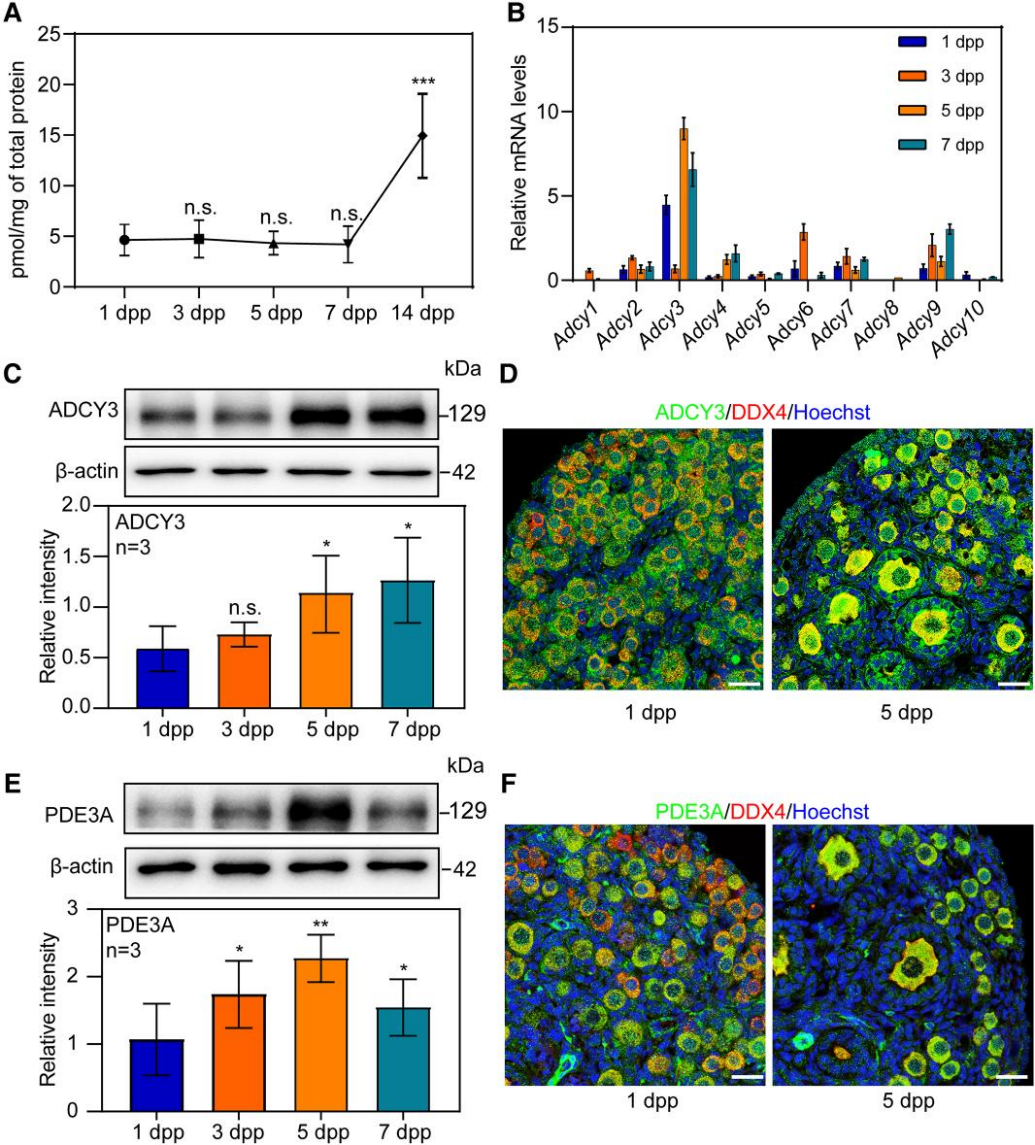
The stable concentration of cAMP is controlled by ADCY3 and PDE3A in neonatal mouse ovaries. (A) cAMP concentration in mouse ovaries was measured from 1 to 14 dpp by ELISA. The concentration of cAMP remained stable from 1 to 7 dpp and then had a significant increase at 14 dpp. (B) The mRNA expressions of all 10 subtypes of Adcys in mouse ovaries were assessed by Real-time PCR. Adcy3 was the dominant subtype during primordial follicle activation, and the mRNA expression level of Adcy3 was increased from 3 to 5 dpp. (C) The total protein level of ADCY3 in neonatal ovaries. Western blotting showed that ADCY3 protein expression significantly increased at 5 dpp. (D) Cellular localization of ADCY3 in mouse ovaries. Neonatal mouse ovaries were stained for ADCY3 and the oocyte marker DDX4 at 1 and 5 dpp. The nuclei were dyed with Hoechst. ADCY3 was localized at the cytoplasm of both oocytes and granulosa cells in either primordial follicles or growing follicles. (E) The total protein level of PDE3A in neonatal ovaries. Western blotting showed that PDE3A protein expression significantly increased at 3 dpp. (F) Cellular localization of PDE3A in mouse ovaries. Neonatal mouse ovaries were stained for PDE3A and DDX4 at 1 and 5 dpp. The nuclei were counter-stained by Hoechst. PDE3A was localized at the cytoplasm of oocytes in follicles. The experiments were repeated at least three times, and representative images were shown. Scale bars: 50 μm.

To investigate the function of cAMP in primordial follicle activation, ELISA assay was employed to detect the concentration of cAMP in the whole mouse ovaries from 1 day post partum (dpp) to 14 dpp. The results showed that the concentration of cAMP in neonatal ovaries remained stable, during the primordial follicle activation was initiated (1–7 dpp). In 14 dpp ovaries, the concentration of cAMP increased significantly (Fig. 1A). These results suggest that cAMP is not only required for follicle formation in mice, as had been approved by our previous work (22) but also may be for follicle growth.

Since the intracellular cAMP concentration is regulated by adenylate cyclases (ADCYs) and phosphodiesterases (PDEs), the two enzymes were then examined in mouse ovaries. Real-time PCR showed that the *Adcy3* was the dominant subtype in neonatal mouse ovaries (Fig. 1B). We further examined the dynamic changes of the ADCY3 protein in neonatal ovaries. It showed that the level of ADCY3 was significantly increased from 1 to 7 dpp, during which time the first wave of primordial follicles activation was initiated (Fig. 1C). Immunofluorescence indicated that the ADCY3 was located in oocytes more than in granulosa cells of primordial and primary follicles (Fig. 1D). These results are broadly in line with the single-cell sequencing data of human ovaries [GSE107746] (23), which revealed the expressions of ADCY3, ADCY5, and ADCY9 were relatively higher than other subtypes in human primordial and primary follicles, and the expressions of the three enzymes were mainly found in the oocytes (Fig. S1A). We next collected and separated the oocytes and somatic cells in 5 dpp mouse ovaries, which contain both primordial follicles and primary follicles (Fig. S1C). The results further proofed that the expression of ADCY3 was higher in oocytes than in granulosa cells (Fig. S1D).

To further uncover the reasons why cAMP remains stable when ADCY3 has such a significant up-regulation in neonatal mouse ovaries, we detected the expression of PDEs in ovaries, which are responsible for the degradation of cAMP. The results showed that the *Pde3a* was the dominant subtype in neonatal mouse ovaries (Fig. S1B). We further noticed that the protein level of PDE3A was up-regulated during the activation of primordial follicles in mouse ovaries (Fig. 1E). Meanwhile, we found that PDE3A was only expressed in the cytoplasm of oocytes of primordial and primary follicles (Fig. 1F, Fig. S1E). Collectively, the expression of ADCY3 and PDE3A contributes to stabilizing the concentration of cAMP harmoniously in neonatal mouse ovaries.

**Fig. 2.**
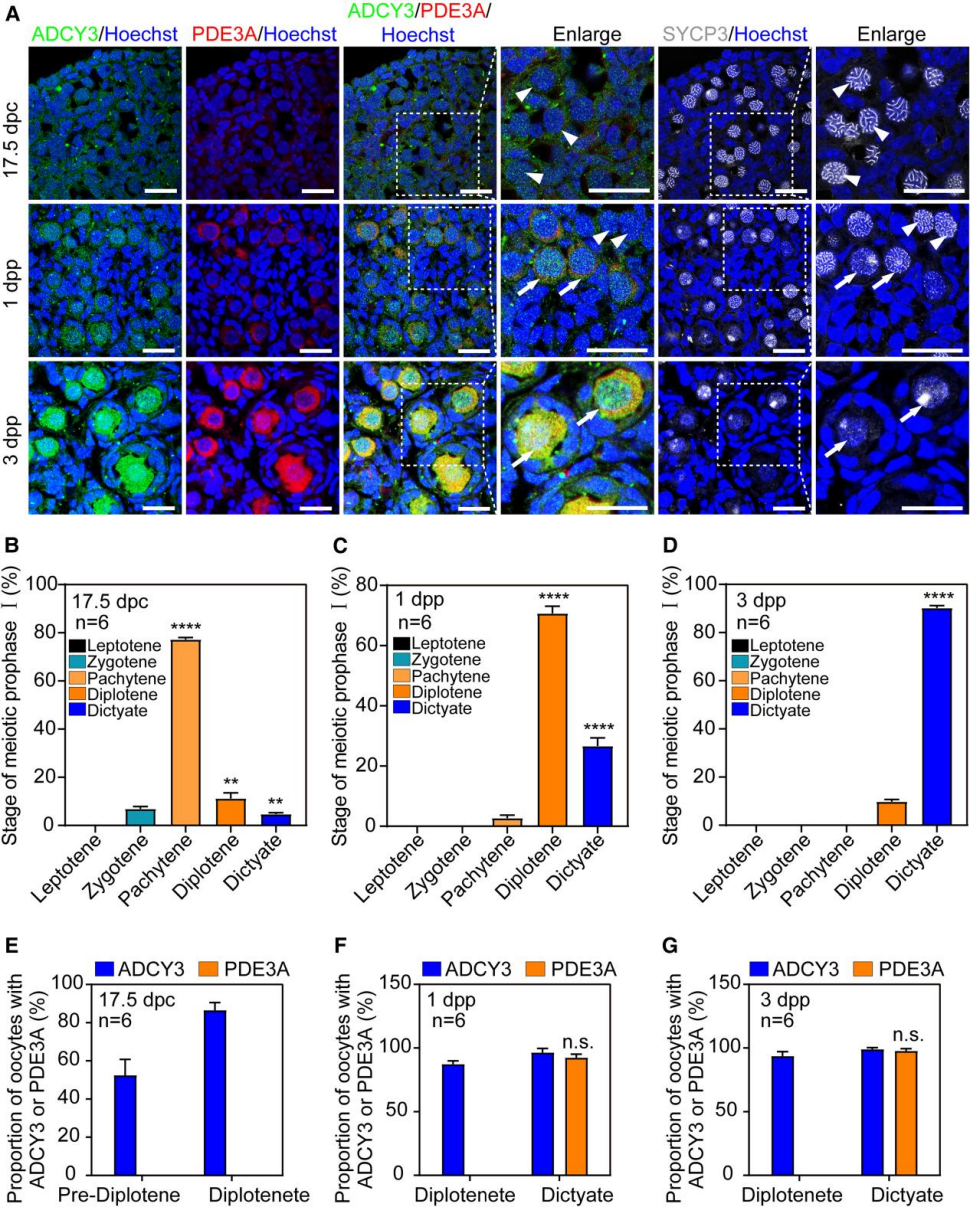
ADCY3 and PDE3A contribute to stabilize the concentration of cAMP in neonatal ovaries. (A) The location of ADCY3 and PDE3A in ovaries at different time points. The nuclei were counter-stained by Hoechst. SYCP3 represented the oocyte meiosis process. ADCY3 was expressed from the prediploid stage (arrowheads) to the dictyate stage (arrows). PDE3A was expressed at dictyate stage (arrows) of primordial follicles. (B–D) Proportion of oocytes in different meiosis stages. Most oocytes have developed to the pachytene stage at 17.5 dpc (B). Most oocytes were undergoing diplotene at 1 dpp (C). Most oocytes were arrested at the dictyate stage at 3 dpp (D). (E–G) The proportion of ADCY3 and PDE3A in oocytes at different time points. Only part of oocytes in prediplotene expressed ADCY3, but almost all diploid oocytes expressed ADCY3 at 17.5 dpc. PDE3A was hardly expressed in oocytes of 17.5 dpc (E). Almost all diploid and dictyate oocytes expressed ADCY3 at 1 and 3 dpp, PDE3A only expressed in dictyate oocytes at 1 and 3 dpp (F, G). The experiments were repeated at least three times, and representative images were shown. Scale bars: 50 μm.

In order to further explore the relationship between ADCY3, PDE3A, and primordial follicle development, we used synaptonemal complex protein 3 (SYCP3) to mark the meiosis process of the oocytes at different time points (Fig. 2A–D), as the development of primordial follicles could be tracked. Then, we performed immunofluorescence co-staining and re-staining to further determine the expression patterns of the ADCY3 and PDE3A in oocytes before and after the formation of primordial follicles. The results showed that ADCY3 was expressed from the prediploid stage (Fig. 2A, arrowheads) to the dictyate stage (Fig. 2A, arrows). However, PDE3A was only expressed in the oocytes arrested at the dictyate stage after primordial follicles were formed (Fig. 2A, arrows). Statistical analysis further confirmed this expression pattern of the ADCY3 and PDE3A (Fig. 2E–G). Taken together, ADCY3 is responsible for the synthesis of cAMP and thus promotes the formation of primordial follicles, whereas the expression of PDE3A becomes necessary only when the primordial follicles were formed in perinatal mouse ovaries. After that, ADCY3 and PDE3A jointly contribute to maintaining the concentration of cAMP at a relatively stable in neonatal mouse ovaries.

### The stable concentration of cAMP plays a regulatory role in the dormancy and activation of primordial follicles in neonatal ovaries

Primordial follicles are gradually formed around birth in mice, and then the first wave of follicles in the medulla area is activated and recruited into the growing follicle pool immediately. Therefore, the neonatal ovaries are ideal models to study the development of the primordial follicle (10, 24). To investigate whether the stability of cAMP would function at the homeostasis of primordial follicle activation in mouse ovaries, we established an in vitro neonatal ovarian culture system to mimic physiological mouse ovarian early development. The histological and statistical analysis results implied that the ovaries cultured in the in vitro system developed normally as they were similar to those in vivo (Fig. S2).

**Fig. 3.**
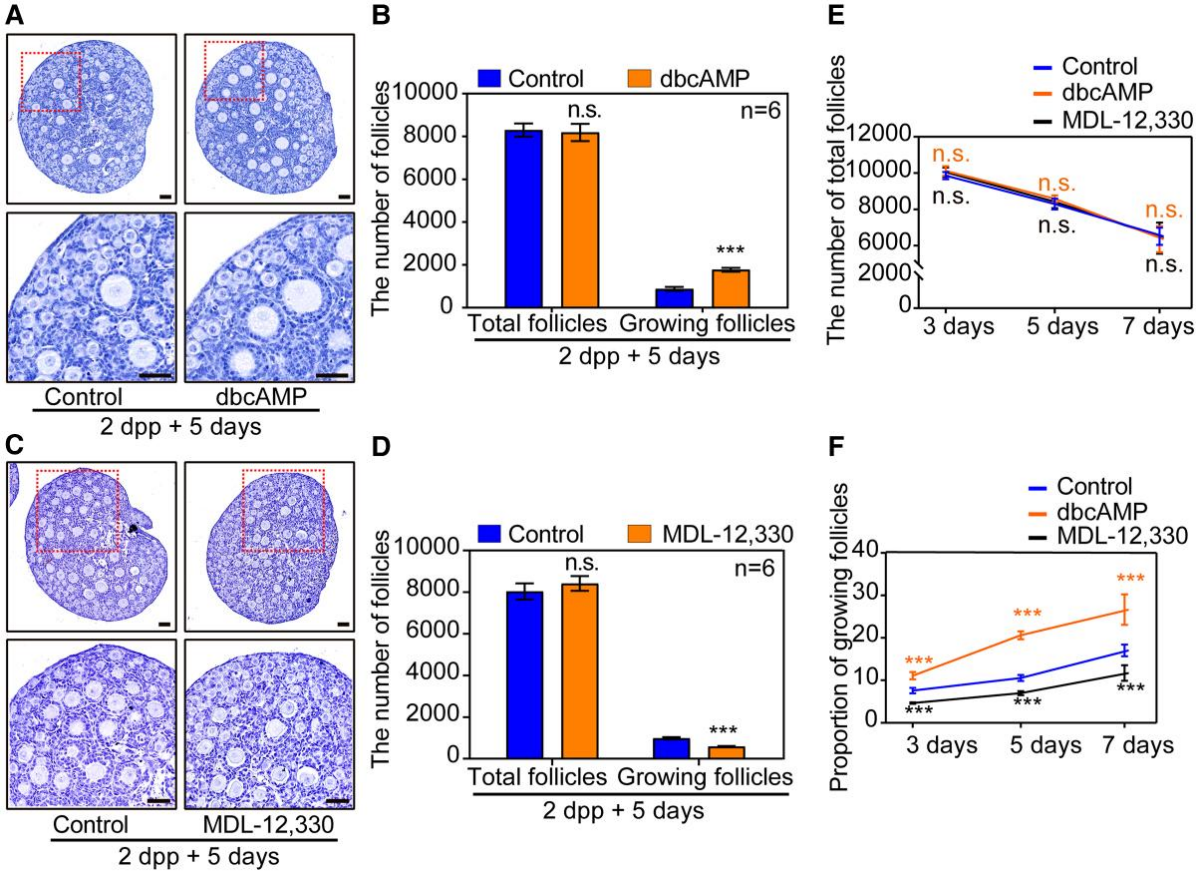
The concentration of cAMP plays a regulatory role in the dormancy and activation of primordial follicles in neonatal ovaries. (A, B) The 2 dpp ovaries were cultured in media alone (control) or with dbcAMP for 5 days in vitro. The histological analysis and follicle counting results showed that the activation of primordial follicles was remarkably increased in dbcAMP-treated ovaries, while the total number of follicles was comparable in control and treated ovaries. (C, D) Ovaries at 2 dpp were cultured without or with MDL-12,330 for 5 days, after inhibition the synthesis of cAMP fewer growing follicles were observed, and quantification of ovarian follicles showed a significant decrease of the growing follicles. (E, F) Follicle quantification in 2 dpp ovaries after 3 days, 5 days, and 7 days cultured showed that dbcAMP significantly promoted primordial follicle activation, while MDL-12,330 remarkably suppressed primordial follicle activation. The total number of follicles in both assays was unaffected. The experiments were repeated at least three times, and representative images were shown. Scale bars: 50 μm.

Based on this system, we cultured 2 dpp ovaries with dibutyryl cAMP (dbcAMP), an analog of cAMP, for 5 days. The results showed that the activation of primordial follicles was accelerated with more growing follicles available in response to dbcAMP induction, while the total number of follicles was comparable to those of the control (Fig. 3A and B). Contrarily, when 2 dpp ovaries were cultured for 5 days with MDL-12,330, an irreversible inhibitor of ADCYs, a remarkable suppression of primordial follicle activation was noticed (Fig. 3C and D). In addition, the number of the total follicles and the growing follicles in ovaries cultured for 3 days and 7 days in either dbcAMP or MDL-12,330 were in line with the 5 days’ results, respectively (Fig. 3E and F). Therefore, the stable concentration of cAMP in neonatal mouse ovaries correlates to the fate of the primordial follicles. This hypothesis was further confirmed by the inhibition of PDE3A in 2 dpp ovaries with Milrinone, which resulted in significant activation of primordial follicles (Fig. S3A and B). Together, the concentration of cAMP plays a regulatory role in the primordial follicle development in neonatal ovaries.

### cAMP is involved in conducting primordial follicle development after puberty

According to the existed report, the second wave of primordial follicles in the cortical area of the mouse ovary starts to be activated at 23 dpp when mouse at the onset of puberty (24).

To validate whether cAMP is involved in the primordial follicle development after puberty, the level of cAMP and the expression of ADCY3 and PDE3A were examined ovaries older than 7 dpp and after puberty. The results suggested that cAMP may play a regulatory role in primordial follicles development after puberty (Fig. S4). After that, the liquid Matrigel containing dbcAMP was injected into the ovarian bursa of 23 dpp mice, which is beneficial for dbcAMP to cover the ovarian surface for a long time and further observe the effect on the follicle development of the ovarian cortex.

**Fig. 4.**
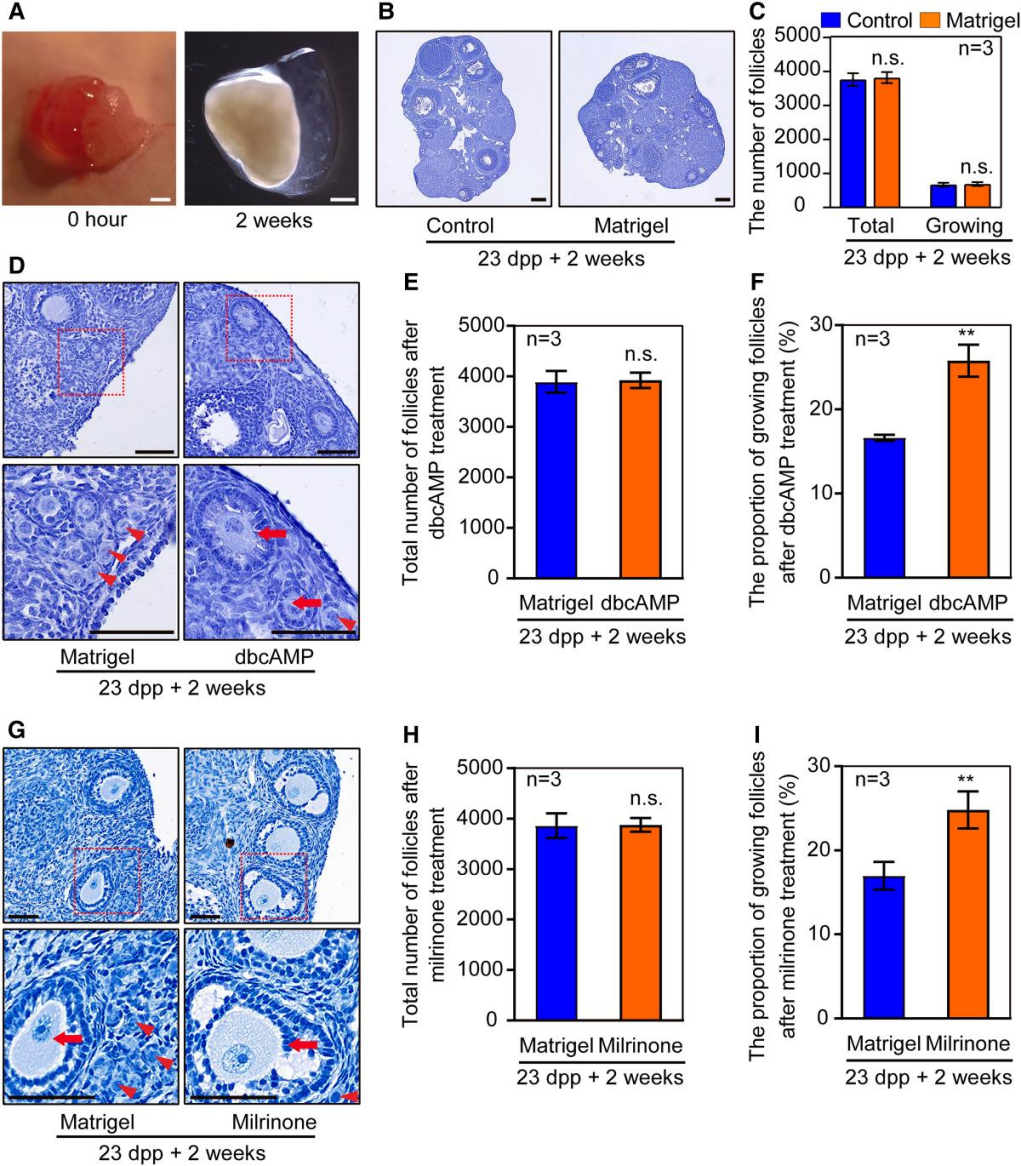
cAMP regulates the activation of primordial follicles after puberty. (A) The Matrigel became solidified in ovarian bursa immediately after injection and was detectable on the ovarian surface 2 weeks after the surgery. (B) The ovarian development was normally 2 after weeks of the surgery. (C) The follicular development was unaffected by Matrigel treatment. (D) A normal distribution of primordial follicles (arrowheads) was observed in the Matrigel ovaries after 2 weeks, whereas an increased number of growing follicles (arrows) was detected in the cortical region of dbcAMP-treated ovaries. (E) dbcAMP treatment had no effect on the total number of follicles. (F) The proportion of growing follicles increased significantly after 2 weeks of dbcAMP treatment. (G–I) Milrinone carried by Matrigel improved the proportion of growing follicles in the cortical region of puberty mice ovaries. The experiments were repeated at least three times, and representative images were shown. Scale bars: 100 μm.

Beforehand, the safety and effectiveness of the Matrigel were verified. We performed ovarian topical administration with Matrigel on 23 dpp mice and measured follicle development after 2 weeks, the results showed that the ovary after the Matrigel treatment was unaffected compared with the contralateral side (Fig. 4A–C). Firstly, the state of the primordial follicles was examined after 2 days of the surgery. Plenty of studies have approved that the un-phosphorylated FOXO3a acts to prevent oocytes from premature activation in primordial follicles. Otherwise, when FOXO3a is phosphorylated (p-FOXO3a) and transferred from the nuclei to the cytoplasm of the oocyte in primordial follicles, the activation of the follicle will be initiated (25–27). The results showed that more oocytes had cytoplasmic localization of FOXO3a (CL-FOXO3a) in the primordial follicles at the ovary cortex in response to dbcAMP induction, as compared to the contralateral control (Fig. S5). Two weeks after the surgery, more growing follicles were observed in the cortical area of the ovary injected with dbcAMP (Fig. 4D, arrows), while most of the primordial follicles remained dormancy in the cortical area of the control group (Fig. 4D, arrowheads). Additionally, the ratio of the growing follicles to the whole follicles in the dbcAMP treatment group was significantly higher than that in the control (Fig. 4F), although there was no difference in total follicle numbers (Fig. 4E). To further confirm the in vivo stimulating effect of cAMP, Milrinone was also dissolved in liquid Matrigel and injected into the ovarian bursas of 23 dpp mice accordingly. The results showed that the activation of primordial follicles in the cortical area of the ovary was significantly accelerated after Milrinone treatment in vivo (Fig. 4G–I). Together, increasing the concentration of cAMP in the ovaries of puberty mice contributes to inducing primordial follicle activation.

### cAMP regulates primordial follicle development by controlling the PI3K and the mTORC1 signaling pathways

**Fig. 5.**
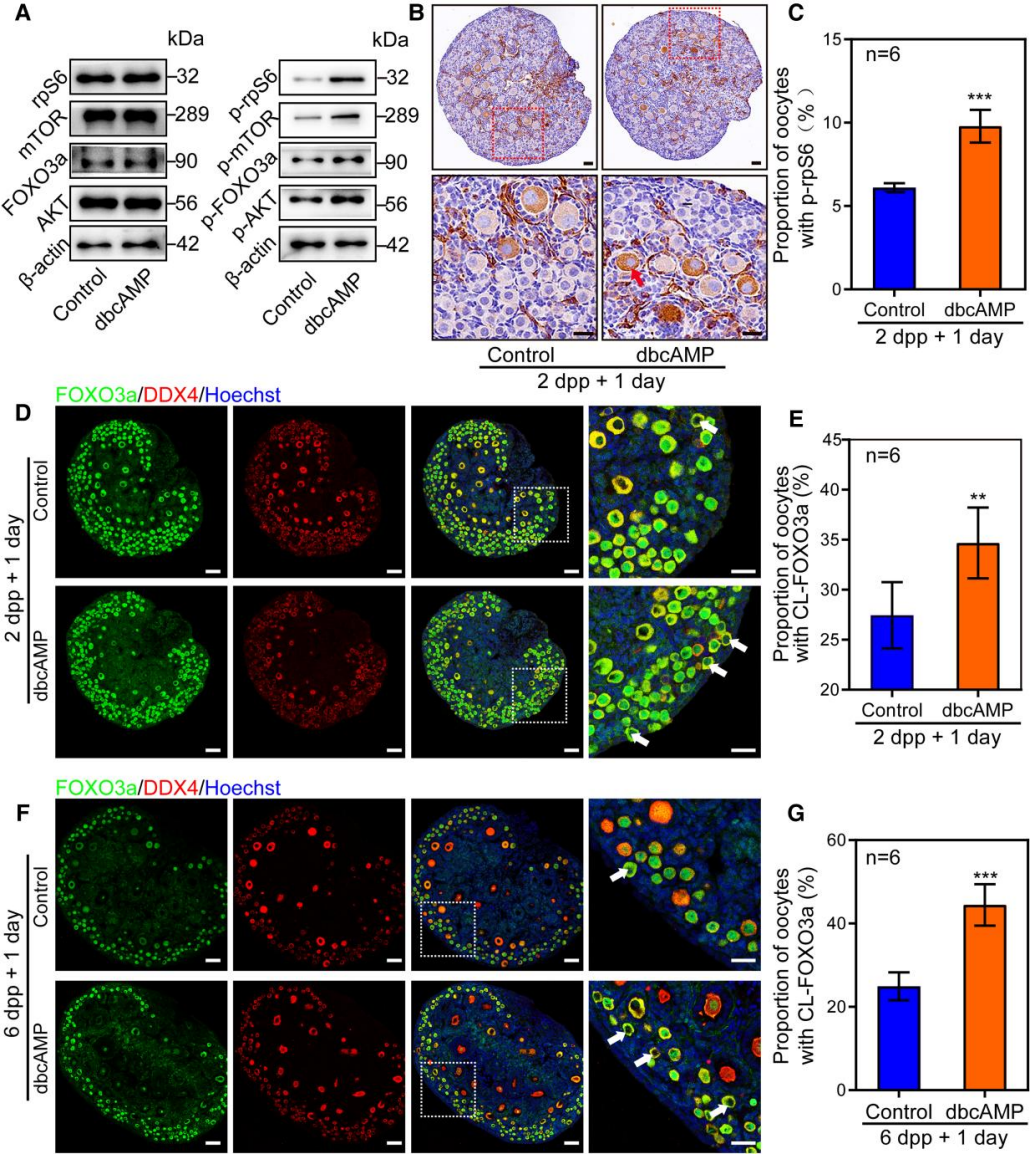
dbcAMP promotes primordial follicle activation via activating the PI3K and the mTOR pathways. (A) The PI3K and mTOR signaling was activated in dbcAMP-treated ovaries. Levels of total AKT, FOXO3a, mTOR, and rpS6 were unchanged compared to controls, but the phosphorylation of these proteins was increased. (B) The oocytes with p-rpS6 positive signals were significantly increased after 2 dpp ovaries were treated with dbcAMP for 1 day. (C) The proportion of oocytes with p-rpS6 positive signals was significantly increased in dbcAMP-cultured ovaries. (D) CL-FOXO3a (arrows) was observed in most of the oocytes after dbcAMP treatment. Oocytes were stained with DDX4. Nuclei were dyed with Hoechst counter-stain. (E) The proportion of CL-FOXO3a was increased after being treated with dbcAMP. (F, G) The proportion of CL-FOXO3a was observably increased after 6 dpp ovaries were cultured with dbcAMP for 1 day. The experiments were repeated at least three times, and representative images were shown. Scale bars: 50 μm.

To evaluate the underlying mechanisms of how cAMP involves in primordial follicle development, the classic signaling cascade, namely the PI3K and mTORC1, were detected. The results showed that the total levels of key proteins in these two signaling pathways were unchanged compared to the controls, whereas the levels of their respective phosphorylation form were all up-regulated by dbcAMP treatment steadily (Fig. 5A). Besides, the number of oocytes with positive signals of phospho-rpS6 (p-rpS6) (Fig. 5B, arrow) was significantly more than the control group after 2 dpp ovaries were treated with dbcAMP for 1 day (Fig. 5C). Meanwhile, after 1 day of culture, the CL-FOXO3a (Fig. 5D, arrows) was remarkably increased (Fig. 5E). In the 2 dpp ovaries, the formation of the primordial follicle pool has not yet been fully accomplished. 6 dpp ovaries with the established primordial follicle pool are selected to verify the effect of the dbcAMP, so as to exclude the side effects of the dbcAMP on the formation of the pool. The effect was repeated by applying dbcAMP on 6 dpp ovaries for 1 day (Fig. 5F and G). These results indicated that dbcAMP promotes primordial follicle activation through the activation of PI3K and mTORC1 signaling pathways.

**Fig. 6.**
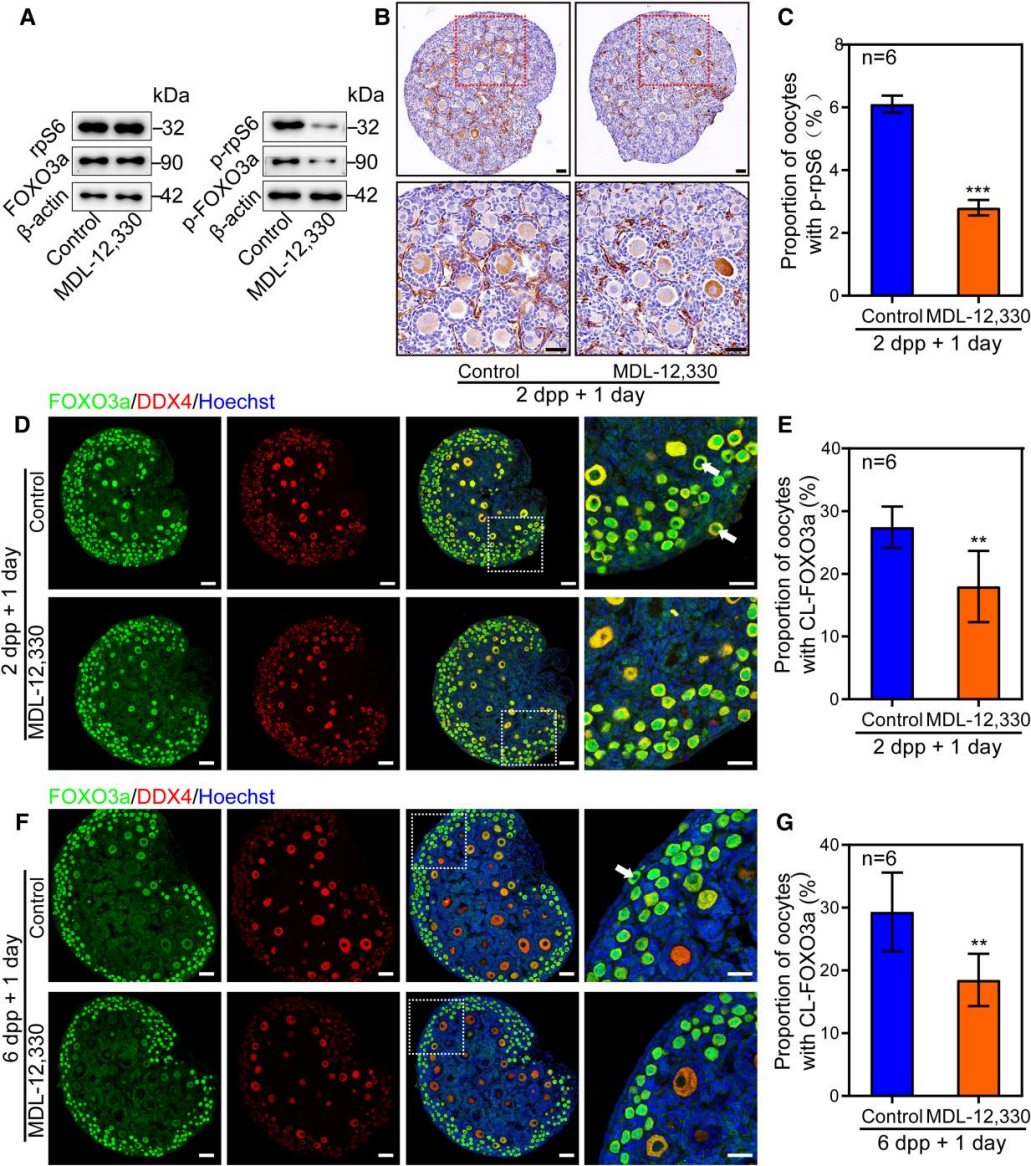
MDL-12,330 suppresses primordial follicle activation via inhibiting the PI3K and the mTOR pathways. (A) Both p-rpS6 and p-FOXO3a were decreased in MDL-12,330 group compared with controls, while the levels of these total proteins were unaffected. (B, C) The oocytes with p-rpS6 positive signals were significantly decreased after 2 dpp ovaries were cultured with MDL-12,330 for 1 day. (D, E) The proportion of CL-FOXO3a was decreased after 2 dpp ovaries were cultured with MDL-12,330 for 1 day. (F, G) The proportion of CL-FOXO3a was decreased after 6 dpp ovaries were cultured with MDL-12,330 for 1 day. The experiments were repeated at least three times, and representative images were shown. Scale bars: 50 μm.

To further approve our hypothesis, we blocked the synthesis of cAMP in cultured mouse ovaries with MDL-12,330. The results showed that dramatic reduction of p-FOXO3a and p-rpS6 expression levels, especially p-rpS6 in oocytes, in treated ovaries were evidenced as compared to the controls (Fig. 6A–C). Besides, fewer oocytes were found to have CL-FOXO3a after MDL-12,330 treatment in both 2 dpp and 6 dpp ovaries (Fig. 6D–G). Inhibiting the degradation of PDE3A by Milrinone increased the CL-FOXO3a as well (Fig. S3C and D). Therefore, cAMP governs primordial follicle development by controlling the activity of PI3K and mTORC1 signaling pathways.

### cAMP controls primordial follicle activation is dependent on protein kinase A

**Fig. 7.**
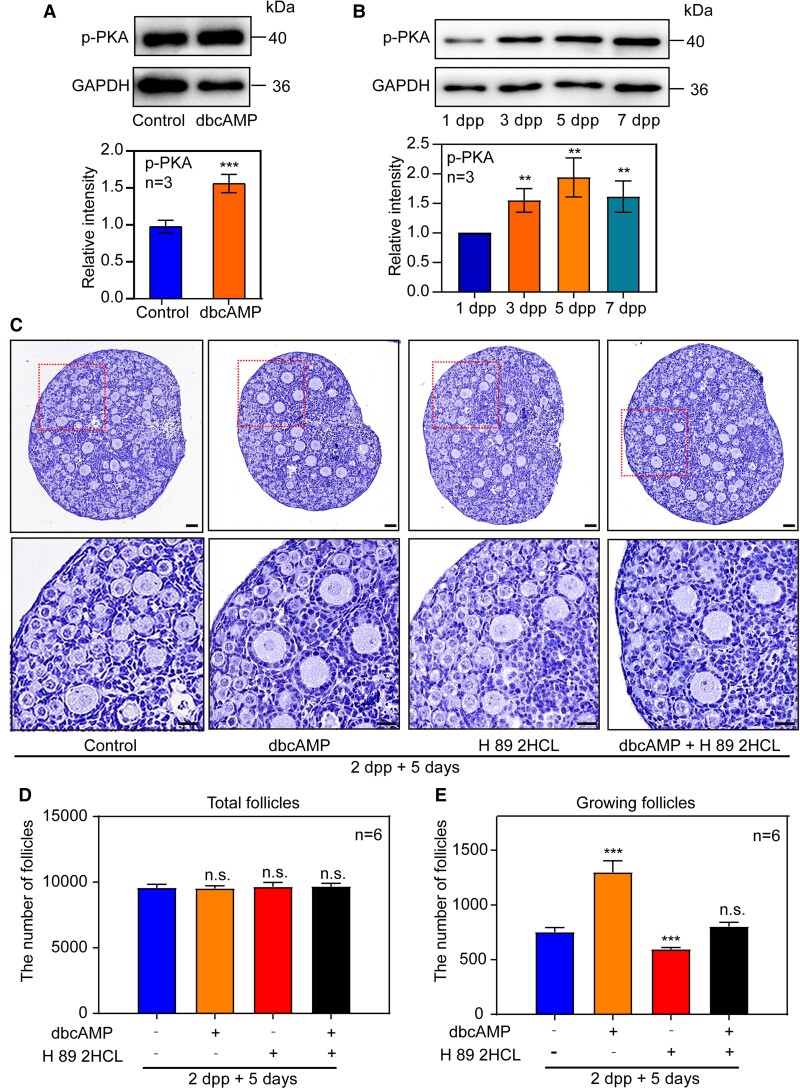
cAMP participates in primordial follicle activation via the action of PKA. (A) p-PKA was increased in dbcAMP group compared with controls after 2 dpp ovaries were cultured for 1 day. (B) The total protein level of p-PKA was increased in neonatal mouse ovaries with primordial follicle activation initialed. (C–E) Ovaries at 2 dpp were cultured in media alone (control) or with dbcAMP or with H 89 2HCL or with dbcAMP plus H 89 2HCL for 5 days in vitro. The primordial follicle activation was increased by dbcAMP and suppressed by H 89 2HCL, further the effect of dbcAMP was reversed by H 89 2HCL treatment. The experiments were repeated at least three times, and representative images were shown. Scale bars: 50 μm.

Generally, cAMP activates protein kinase A (PKA) and phosphorylates specific proteins to achieve signal transduction in cells. We found that phosphorylated PKA (p-PKA) was up-regulated after 1 day of dbcAMP treatment, beginning at 2 dpp, compared to controls (Fig. 7A). In addition, both the results of Milrinone and MDL-12,330 treatment, together with the results of examined after puberty provide evidences confirming that PKA may play a role in follicle activation as the downstream of cAMP (Fig. S6).

**Fig. 8.**
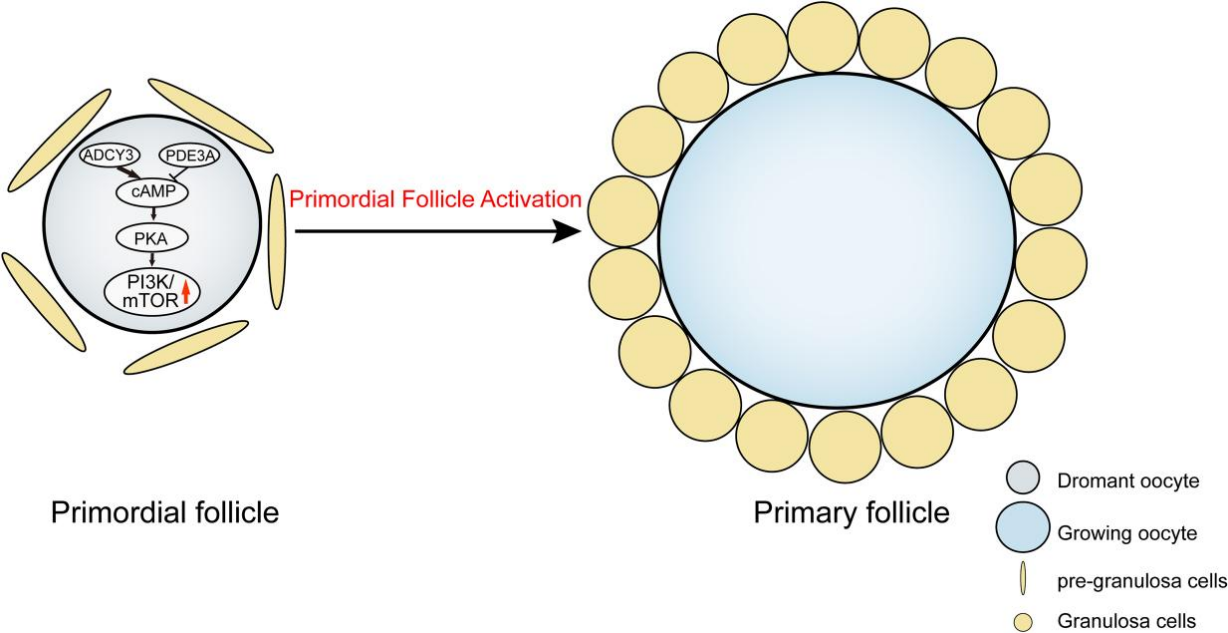
The diagram of cAMP promotes the activation of primordial follicle. cAMP in dormant oocyte via PKA activates PI3K/mTOR signaling pathways, thus promoting primordial follicle activation.

Surprisingly, the expression of p-PKA was increased during the primordial follicle initial activation in neonatal mouse ovaries (Figs. 7B and S7). To further confirmed that cAMP regulates primordial follicle activation and is dependent on PKA, 2 dpp ovaries were cultured for 5 days with or without H 89 2HCL, an effective inhibitor of PKA, was used to verify if it could reverse the excessive activation of primordial follicles caused by dbcAMP. The histological analysis and whole ovary counting results showed that fewer growing follicles were observed in the dbcAMP plus H 89 2HCL treatment than those in the dbcAMP treatment alone (Fig. 7C–E). Interestingly, fewer growing follicles were observed in H 89 2HCL alone treatment compared to the control. After that, the activation of the mTORC1/PI3K signaling pathways was suppressed by H 89 2HCL (Fig. S8). Collectively, these results confirmed that the cAMP–PKA–mTORC1/PI3K pathway participates in regulating the development of primordial follicles (Fig. 8).

## Discussion

Primordial follicles, as the most abundant fertility resource in females, can progress further development and contribute to female fertility only when they have been activated (28). cAMP has been proved to regulate the proliferation of mouse primordial germ cells and the differentiation of granulosa cells more than 20 years ago (29, 30). The most important role of cAMP in oogenesis is its relationship with the process of meiosis so far. In fetal mouse ovaries, the concentration of cAMP is increased when oocyte enters meiosis prophase I (22). More importantly, immature oocytes in antral follicles are arrested steadily at the dictyate stage due to the concentration of cAMP being strictly maintained at a relatively higher concentration (20, 31). However, the role of cAMP during the development of primordial follicles is not clear until now. In this study, the important role of cAMP in the dormancy and activation of mouse primordial follicles is emphasized. The concentration of cAMP remains stable in neonatal mouse ovaries, which may aim to balance the speed of primordial follicle activation in physiological conditions. The findings not only re-emphasize the idea that cAMP has an irreplaceable role in the female reproductive system (Fig. S9) but also supply proofs to deeper understand the underlined mechanisms of balancing the activation and dormancy of primordial follicles. The concentration of intracellular cAMP is regulated by the synthetase ADCYs and the hydrolytic enzyme PDEs (32–34). Horner et al. pointed out that ADCY3 may further participate in the process of meiosis by regulating the synthesis of cAMP in rodent oocytes (35). So far, PDE3A is mainly responsible for the hydrolyzing of cAMP in oocytes. PDE3A-deficient oocytes exhibit severe cell cycle arrest, which eventually causes infertility in mice (36). In agreement with these findings, we found that both ADCY3 and PDE3A are up-regulated in neonatal mouse ovaries during the activation of primordial follicles, which is responsible for maintaining the stability of cAMP concentration. Nevertheless, the possible functions of the rest ADCYs and PDEs in the primordial follicles of ovaries need further study.

The primordial follicle activation requires complex and orchestrated adjustment of oocytes and granulosa cells (3). In adult female ovaries, the levels of mTORC1 signaling activity in granulosa cells through KITL-KIT control the PI3K signaling in oocytes, thus deciding whether or not a dormant oocyte will be awaked (8, 14, 37). The most recent findings have shown that the first wave of primordial follicle activation may be initiated by oocyte-originated signals (38). According to our findings, cAMP in oocytes may be pivotal for the first wave of follicles as ADCY3 and PDE3A are mainly expressed in the oocytes of primordial follicles and primary follicles in neonatal ovaries. On one hand, cAMP significantly stimulates the expression of KITL in granulosa cells at the early folliculogenesis (39, 40). On the other hand, it may directly or indirectly regulate the oocyte-specific gene expression KIT, NOBOX, GDF9, and BMP15 which are indispensable in the activation of primordial follicles (37, 41, 42). Therefore, the role of cAMP in granulosa cells cannot be ruled out, and more suitable animal models are needed for identifying its action in the future.

The signals of extracellular bind to G protein-coupled receptors (GPRs) on the cell membrane and stimulate the concentration of cAMP of intracellular. So, whether signals in the ovarian microenvironment depend on GPRs to stimulate cAMP synthesis in oocytes or granulosa cells, and further involve in the development of primordial follicles is not clear as well. It has been reported that the concentration of cAMP required to maintain meiosis arrest of mouse and rat oocytes depends on GPR3 or GPR12, respectively (43). The resumption of meiosis would be activated after GPR3 knockout and causes premature ovarian failure (44). However, the role of GPRs in primordial follicle activation is still not clear. In addition to the endogenous synthesis, the concentration of cAMP in cells could also be transported from other cells. Whether the high concentration of cAMP in growing follicles would participate in regulating the development of the surrounding primordial follicles by transporting cAMP needs to be further explored.

PKA as a direct target of cAMP participates in regulating various physiological processes by regulating gene expression (45, 46). The role of cAMP as the second messenger of gonadotropin in oocyte maturation is also mediated by PKA (46). Here, we found that cAMP controls primordial follicle activation and is dependent on PKA as well. Some studies have found that the cAMP/PKA pathway interacts with PI3K/AKT and mTORC1 signaling pathways in ovaries and other tissues (47–49). However, the relationship between cAMP/PKA with PI3K/AKT and mTORC1 signaling pathways in primordial follicle dormancy and activation needs further study.

The dbcAMP or Milrinone might be the safe and efficient candidate medicines for the improvement of in vitro activation (IVA) of primordial follicles. Helping primary ovarian failure patients to have their own babies through IVA techniques is becoming practicable since the technique has been established (28, 50). The present targets for achieving IVA focus on mTORC1 and PI3K in present clinic practice, which has certain limitations (51). Our study, however, provides cAMP as a novel candidate target for achieving IVA in the future. Milrinone, which could effectively inhibit the degradation of cAMP, has been reported clinically for treating heart failure and other diseases (52–54), which implies the safety of the chemical has been concerned, supervised, and approved. We found that Milrinone is effective to activate primordial follicles both in vitro and in vivo, which implies that applying Milrinone to target cAMP could be effective in clinic. However, more IVA trials and animal models are needed before Milrinone could be accepted as one of the alternative choices.

In summary, sustaining the concentration of cAMP in the ovary at an appropriate concentration is vital for the balance between dormancy and activation of mouse primordial follicles. Targeting cAMP as one of the candidate molecules to perform IVA in clinic could be one of the plausible choices.

## Materials and methods

### Animals

All CD-1 mice were obtained from Beijing Vital River Laboratory Animal Technology Co., Ltd. and housed in China Agricultural University with 12 h light/12 h dark cycles, the temperature at 24–26 °C, with free access to food and water. We always consider that mice with a vaginal plug in the next morning of mated as 0.5 day post coitus (dpc). The day after birth was considered as 1 dpp. All procedures and facilities were conducted in accordance with the guidelines of and approved by the Animal Research Committee of the China Agricultural University (Beijing, China).

### In vitro culture of mouse ovaries

A safe and effective in vitro ovarian culture system has been established in our lab (10, 11). Neonatal mice were sacrificed by cervical dislocation at the designated times. The ovaries were separated in cold phosphate-buffered saline (PBS) under the microscope. The isolated ovaries were incubated in 6-well culture dishes (NEST, China), and an insert (PICM0RG50, Millipore, USA) was placed in every well with 3 mL Dulbecco Modified Eagle Medium/Ham F12 nutrient mixture (DMEM/F12) (Gibco, Life Technologies, CA) supplemented with insulin-transferrin-sodium selenite (1:100, Sigma, USA) and penicillin-streptomycin solution at 37 °C, 5% CO_2_ and saturated humidity. Ovaries were randomly assigned, and cultured for 1–7 days in basal medium alone or basal medium supplemented with either dbcAMP (10 μM, D0627, Sigma, USA), MDL-12,330 (5 μM, M182, Sigma, USA), Milrinone (10 μM, 78415-72-2, J&K, China) or H 89 2HCL (5 μM, S1582, Selleck, China), respectively.

### Ovarian topical administration in vivo

According to Zhang's protocol (55), female mice were anesthetized with avertin (300 mg/kg, T48402, Sigma, USA) before surgery. In the control group, the precooled growth factor reduced Matrigel (354230, BD, USA) was injected into the unilateral ovarian bursa using an insulin syringe. The contralateral ovary was injected with the Matrigel containing either dbcAMP or Milrinone, accordingly. After the temperature-sensitive Matrigel was solidified, the incisions were sutured.

### Enzyme-linked immunosorbent assay (ELISA)

The concentration of cAMP present in mouse ovaries was measured with the Cyclic AMP Direct Chemiluminescent ELISA Kit (K019-C1, Arbor Assays, USA). 5 mg ovaries were collected at each time point in a 1.5 mL centrifuge tube, the ovaries should be frozen in liquid nitrogen and stored at −80 °C, if the analysis is not to be carried out immediately. Before analysis, grind the frozen tissue under a low temperature until it is a fine powder. Add 100 μL of Sample Diluent to every tube. Incubate in the Sample Diluent for 10 min on ice, and then centrifuge at 800*g* for 15 min. The supernatant was collected, and cAMP concentration was measured based on the manufacturer's instructions and expressed as picomoles per milligram of tissue. The protein concentration was measured using the BCA protein assay kit (P0010, Beyotime Biotechnology, China) using bovine serum albumin (BSA) as the standard. The values were further quantified as protein (mg) present in the sample.

### Histological analysis and follicle counting

Samples of mouse ovaries were collected and fixed with 4% PFA at 4 °C overnight, dehydrated in gradient alcohol, embedded in paraffin, and performed to 5 μm serial sections. The ovarian sections after deparaffinized were stained with hematoxylin. Based on the well-accepted standards established by Pedersen and Peters, every section was counted for the presence of primordial (type 2), primary (type 3), secondary (type 4 and 5), and antral (types 6 and 7) follicles (56). Only when follicles with the clearly visible nucleus of oocytes were counted on each slide. We recorded the primary, secondary, and antral follicles as growing follicles in statistical analysis.

### Immunofluorescence and immunohistochemistry

The ovarian paraffin sections were deparaffinized, rehydrated, and subjected to high temperature (95–98 °C) antigen retrieval with 0.01% sodium citrate buffer (pH 6.0) for 16 min. After that, the sections were washed with PBS on a shaker for 5 min, and then blocked with 1% normal donkey serum in PBS for 1 h at room temperature and incubated with primary antibodies for 12–16 h at 4 °C. The antibodies used were listed in Table S1. Subsequently, ovarian sections were rinsed thoroughly with PBS and incubated with Alexa Fluor 488- or 555-conjugated donkey secondary antibodies for 1 h at 37 °C. Then slides were rinsed in PBS, counter-stained with Hoechst 33342 for 5 min. Finally, sealed the sections in an anti-fade fluorescence mounting medium with coverslips. Immunohistochemistry was performed using Histostain™-SP Kits (PV-9001, ZSGB-BIO, China) and DAB peroxidase substrate kits (ZLI-9017, ZSGB-BIO, China) according to the manufacturer's protocols. Sections were examined and photographed using Nikon 80i or Nikon A1 laser.

### Immunofluorescence re-staining

In this study, both anti-ADCY3 and anti-PDE3A are rabbit IgG antibodies, we performed immunofluorescence re-staining to observe their localization and expression in ovaries (34). After the immunofluorescence staining of ADCY3, the position of the section was recorded when taking photos. Subsequently, the original primary and secondary antibodies on the sections were eluted with stripping buffer (P0025, Beyotime Biotechnology, China), and then PDE3A was re-stained according to immunofluorescence staining protocols. Finally, photos were taken at the same location.

### Western blotting analysis

The ovarian samples were lysed in WIP lysis solution (8553S, Cell Signaling Technologies, USA). Sample proteins were separated by electrophoresis by 7.5% SDS-PAGE and then transferred to PVDF (polyvinylidene fluoride) membranes (IPVH00010, Millipore, USA). The membranes were blocked with 5% nonfat-dry milk for 1 h at room temperature and incubated at 4 °C overnight with appropriate primary antibodies. The antibodies used were listed in Table S1. After that, the membranes were washed thoroughly with tris buffered saline tween (TBST) and incubated with the secondary antibody (1:4,000, ZSGB-BIO, China) for 1 h at 37 °C and rinsed thoroughly with TBST. The membranes were visualized by the SuperSignal detection system (Prod 34080, Thermo, USA). To quantify the results of immunoblot, the image was quantified using Image J software.

### Real-time PCR analysis

The ovarian samples were extracted by TRIZOL Reagent (Invitrogen, Life Technologies, USA) according to the manufacturer's protocol. First-strand cDNA was synthesized by reverse transcription using 1 µg of total RNA (Promega Reverse Transcription System, Promega, USA). Quantitative RT-PCR reactions were operated and analyzed by LightCycler 96 Real-Time PCR System (Roche, Switzerland). Data were normalized by *β-actin*. Primers were listed in Table S2.

### Adherent separation of oocytes and somatic cells in neonatal mouse ovaries

Twenty ovaries were collected in a centrifuge tube, followed by complete digestion with the addition of 0.125% pancreatin preheated at 37 °C, and the single-cell suspension was transferred to stop solution of the same volume, then centrifuge at 3,000 rpm for 5 min. The sediment is the ovarian cells. Resuspend ovarian cells with 1200 μL DMEM/F12 containing 10% FBS, and cultured them in a 6-well culture dish (COAST, USA) at 37 °C, 5% CO_2_, and saturated humidity for 4–6 h. Until most of the somatic cells grow adherently (spindle-shaped), while the oocytes are still suspended (round-shaped), collecting the cells in centrifuge tubes, respectively.

### Statistical analysis

All experiments were repeated at least three times, and the data were presented as the means ± SEM, analyzed by *t* test. Statistically significant values of *P* < 0.05, *P* < 0.01, and *P* < 0.001 are indicated by asterisks (*), (**), and (***), respectively.

## Supplementary Material

pgad055_Supplementary_Data

## Data Availability

All data are included in the manuscript and/or supporting information.

## References

[1] McGee EA , Hsueh AJ. 2000. Initial and cyclic recruitment of ovarian follicles. Endocr Rev. 21:200–214. 10782364 10.1210/edrv.21.2.0394

[2] Zhang H , et al 2014. Life-long in vivo cell-lineage tracing shows that no oogenesis originates from putative germline stem cells in adult mice. Proc Natl Acad Sci U S A. 111:17983–17988. 25453063 10.1073/pnas.1421047111 PMC4273382

[3] Adhikari D , Liu K. 2009. Molecular mechanisms underlying the activation of mammalian primordial follicles. Endocr Rev. 30:438–464. 19589950 10.1210/er.2008-0048

[4] Zhang H , et al 2015. Adult human and mouse ovaries lack DDX4-expressing functional oogonial stem cells. Nat Med. 21:1116–1118. 26444631 10.1038/nm.3775

[5] Qin Y , Jiao X, Simpson JL, Chen Z-J. 2015. Genetics of primary ovarian insufficiency: new developments and opportunities. Hum Reprod Update. 21:787–808. 26243799 10.1093/humupd/dmv036 PMC4594617

[6] Reddy P , et al 2008. Oocyte-specific deletion of Pten causes premature activation of the primordial follicle pool. Science. 319:611–613. 18239123 10.1126/science.1152257

[7] Reddy P , et al 2009. PDK1 signaling in oocytes controls reproductive aging and lifespan by manipulating the survival of primordial follicles. Hum Mol Genet. 18:2813–2824. 19423553 10.1093/hmg/ddp217

[8] Zhang H , et al 2014. Somatic cells initiate primordial follicle activation and govern the development of dormant oocytes in mice. Curr Biol. 24:2501–2508. 25438940 10.1016/j.cub.2014.09.023

[9] Ren Y , et al 2015. Lhx8 regulates primordial follicle activation and postnatal folliculogenesis. BMC Biol. 13:39. 26076587 10.1186/s12915-015-0151-3 PMC4487509

[10] Yan H , et al 2018. CDC42 controls the activation of primordial follicles by regulating PI3K signaling in mouse oocytes. BMC Biol. 16:73. 29976179 10.1186/s12915-018-0541-4 PMC6033292

[11] Zhang T , et al 2019. SIRT1 facilitates primordial follicle recruitment independent of deacetylase activity through directly modulating Akt1 and mTOR transcription. FASEB J. 33:14703–14716. 31693862 10.1096/fj.201900782R

[12] Yan H , et al 2019. Oocyte-derived E-cadherin acts as a multiple functional factor maintaining the primordial follicle pool in mice. Cell Death Dis. 10:160. 30770786 10.1038/s41419-018-1208-3 PMC6377673

[13] Zhang T , et al 2021. HDAC6 regulates primordial follicle activation through mTOR signaling pathway. Cell Death Dis. 12:559. 34052832 10.1038/s41419-021-03842-1 PMC8164630

[14] Zhang H , Liu K. 2015. Cellular and molecular regulation of the activation of mammalian primordial follicles: somatic cells initiate follicle activation in adulthood. Hum Reprod Update. 21:779–786. 26231759 10.1093/humupd/dmv037

[15] Nilsson EE , Skinner MK. 2004. Kit ligand and basic fibroblast growth factor interactions in the induction of ovarian primordial to primary follicle transition. Mol Cell Endocrinol. 214:19–25. 15062541 10.1016/j.mce.2003.12.001

[16] Zhang F , Zhang L, Qi Y, Xu H. 2016. Mitochondrial cAMP signaling. Cell Mol Life Sci. 73:4577–4590. 27233501 10.1007/s00018-016-2282-2 PMC5097110

[17] Hernandez-Ramirez LC , Trivellin G, Stratakis CA. 2018. Cyclic 3′,5′-adenosine monophosphate (cAMP) signaling in the anterior pituitary gland in health and disease. Mol Cell Endocrinol. 463:72–86. 28822849 10.1016/j.mce.2017.08.006

[18] Cho WK , Stern S, Biggers JD. 1974. Inhibitory effect of dibutyryl cAMP on mouse oocyte maturation in vitro. J Exp Zool. 187:383–386. 4362350 10.1002/jez.1401870307

[19] Mehlmann LM , et al 2004. The Gs-linked receptor GPR3 maintains meiotic arrest in mammalian oocytes. Science. 306:1947–1950. 15591206 10.1126/science.1103974

[20] Conti M , Hsieh M, Zamah AM, Oh JS. 2012. Novel signaling mechanisms in the ovary during oocyte maturation and ovulation. Mol Cell Endocrinol. 356:65–73. 22101318 10.1016/j.mce.2011.11.002 PMC4104635

[21] Zhang M , Su YQ, Sugiura K, Xia G, Eppig JJ. 2010. Granulosa cell ligand NPPC and its receptor NPR2 maintain meiotic arrest in mouse oocytes. Science. 330:366–369. 20947764 10.1126/science.1193573 PMC3056542

[22] Wang Y , et al 2015. Cyclic AMP in oocytes controls meiotic prophase I and primordial folliculogenesis in the perinatal mouse ovary. Development. 142:343–351. 25503411 10.1242/dev.112755

[23] Zhang Y , et al 2018. Transcriptome landscape of human folliculogenesis reveals oocyte and granulosa cell interactions. Mol Cell. 72:1021–1034.e1024. 30472193 10.1016/j.molcel.2018.10.029

[24] Zheng W , et al 2014. Two classes of ovarian primordial follicles exhibit distinct developmental dynamics and physiological functions. Hum Mol Genet. 23:920–928. 24087793 10.1093/hmg/ddt486 PMC3900105

[25] Zhang X , et al 2022. Enhanced glycolysis in granulosa cells promotes the activation of primordial follicles through mTOR signaling. Cell Death Dis. 13:87. 35087042 10.1038/s41419-022-04541-1 PMC8795455

[26] Chang EM , et al 2015. Cisplatin induces overactivation of the dormant primordial follicle through PTEN/AKT/FOXO3a pathway which leads to loss of ovarian reserve in mice. Plos One. 10:e0144245. 10.1371/journal.pone.0144245 PMC4699462 26656301

[27] Jang H , et al 2017. Synergistic effect of melatonin and ghrelin in preventing cisplatin-induced ovarian damage via regulation of FOXO3a phosphorylation and binding to the p27(Kip1) promoter in primordial follicles. J Pineal Res. 63:e12432. 10.1111/jpi.12432 28658519

[28] Kawamura K , Kawamura N, Hsueh AJ. 2016. Activation of dormant follicles: a new treatment for premature ovarian failure? Curr Opin Obstet Gynecol. 28:217–222. 27022685 10.1097/GCO.0000000000000268 PMC5536116

[29] De Felici M , Dolci S, Pesce M. 1993. Proliferation of mouse primordial germ cells in vitro: a key role for cAMP. Dev Biol. 157:277–280. 8387035 10.1006/dbio.1993.1132

[30] Grieshaber NA , Boitano S, Ji IH, Mather JP, Ji TH. 2000. Differentiation of granulosa cell line: follicle-stimulating hormone induces formation of lamellipodia and filopodia via the adenylyl cyclase/cyclic adenosine monophosphate signal. Endocrinology. 141:3461–3470. 10965919 10.1210/endo.141.9.7654

[31] He M , Zhang T, Yang Y, Wang C. 2021. Mechanisms of oocyte maturation and related epigenetic regulation. Front Cell Dev Biol. 9:654028. 10.3389/fcell.2021.654028 PMC8025927 33842483

[32] Sunahara RK , Dessauer CW, Gilman AG. 1996. Complexity and diversity of mammalian adenylyl cyclases. Annu Rev Pharmacol Toxicol. 36:461–480. 8725398 10.1146/annurev.pa.36.040196.002333

[33] Arshavsky VY , Lamb TD, Pugh EN Jr. 2002. G proteins and phototransduction. Annu Rev Physiol. 64:153–187. 11826267 10.1146/annurev.physiol.64.082701.102229

[34] Wettschureck N , Offermanns S. 2005. Mammalian G proteins and their cell type specific functions. Physiol Rev. 85:1159–1204. 16183910 10.1152/physrev.00003.2005

[35] Horner K , et al 2003. Rodent oocytes express an active adenylyl cyclase required for meiotic arrest. Dev Biol. 258:385–396. 12798295 10.1016/s0012-1606(03)00134-9

[36] Begum N , Shen W, Manganiello V. 2011. Role of PDE3A in regulation of cell cycle progression in mouse vascular smooth muscle cells and oocytes: implications in cardiovascular diseases and infertility. Curr Opin Pharmacol. 11:725–729. 22051884 10.1016/j.coph.2011.10.006 PMC3225595

[37] Driancourt MA , Reynaud K, Cortvrindt R, Smitz J. 2000. Roles of KIT and KIT LIGAND in ovarian function. Rev Reprod. 5:143–152. 11006164 10.1530/ror.0.0050143

[38] Dai YL , et al 2022. Asynchronous embryonic germ cell development leads to a heterogeneity of postnatal ovarian follicle activation and may influence the timing of puberty onset in mice. BMC Biol. 20:109. 35550124 10.1186/s12915-022-01318-y PMC9101839

[39] Yao K , Ge W. 2015. Differential regulation of kit ligand A (kitlga) expression in the zebrafish ovarian follicle cells—evidence for the existence of a cyclic adenosine 3′, 5′ monophosphate-mediated binary regulatory system during folliculogenesis. Mol Cell Endocrinol. 402:21–31. 25542847 10.1016/j.mce.2014.12.005

[40] Liu J-C , et al 2021. Di (2-ethylhexyl) phthalate impairs primordial follicle assembly by increasing PDE3A expression in oocytes. Environ Pollut. 270:116088. 10.1016/j.envpol.2020.116088 33234378

[41] Rajkovic A , Pangas SA, Ballow D, Suzumori N, Matzuk MM. 2004. NOBOX deficiency disrupts early folliculogenesis and oocyte-specific gene expression. Science. 305:1157–1159. 15326356 10.1126/science.1099755

[42] Yan C , et al 2001. Synergistic roles of bone morphogenetic protein 15 and growth differentiation factor 9 in ovarian function. Mol Endocrinol. 15:854–866. 11376106 10.1210/mend.15.6.0662

[43] Hinckley M , Vaccari S, Horner K, Chen R, Conti M. 2005. The G-protein-coupled receptors GPR3 and GPR12 are involved in cAMP signaling and maintenance of meiotic arrest in rodent oocytes. Dev Biol. 287:249–261. 16229830 10.1016/j.ydbio.2005.08.019

[44] Ledent C , et al 2005. Premature ovarian aging in mice deficient for Gpr3. Proc Natl Acad Sci U S A. 102:8922–8926. 15956199 10.1073/pnas.0503840102 PMC1150279

[45] Montminy M . 1997. Transcriptional regulation by cyclic AMP. Annu Rev Biochem. 66:807–822. 9242925 10.1146/annurev.biochem.66.1.807

[46] Dey S , Brothag C, Vijayaraghavan S. 2019. Signaling enzymes required for sperm maturation and fertilization in mammals. Front Cell Dev Biol. 7:341. 31921853 10.3389/fcell.2019.00341 PMC6930163

[47] Chen Y-J , et al 2007. Interplay of PI3K and cAMP/PKA signaling, and rapamycin-hypersensitivity in TGFbeta1 enhancement of FSH-stimulated steroidogenesis in rat ovarian granulosa cells. J Endocrinol. 192:405–419. 17283241 10.1677/JOE-06-0076

[48] Zhao L-X , et al 2019. M1 muscarinic receptors regulate the phosphorylation of AMPA receptor subunit GluA1 via a signaling pathway linking cAMP–PKA and PI3K-Akt. FASEB J. 33:6622–6631. 30794430 10.1096/fj.201802351R

[49] Yang Y , et al 2021. The imbalance of PGD2-DPs pathway is involved in the type 2 diabetes brain injury by regulating autophagy. Int J Biol Sci. 17:3993–4004. 34671214 10.7150/ijbs.60149 PMC8495389

[50] Kawamura K , et al 2013. Hippo signaling disruption and Akt stimulation of ovarian follicles for infertility treatment. Proc Natl Acad Sci U S A. 110:17474–17479. 24082083 10.1073/pnas.1312830110 PMC3808580

[51] Sun X , et al 2015. New strategy for in vitro activation of primordial follicles with mTOR and PI3K stimulators. Cell Cycle. 14:721–731. 25590233 10.1080/15384101.2014.995496 PMC4615062

[52] Felker GM , et al 2003. Heart failure etiology and response to milrinone in decompensated heart failure—results from the OPTIME-CHF study. J Am Coll Cardiol. 41:997–1003. 12651048 10.1016/s0735-1097(02)02968-6

[53] Chong LYZ , Satya K, Kim B, Berkowitz R. 2018. Milrinone dosing and a culture of caution in clinical practice. Cardiol Rev. 26:35–42. 29045285 10.1097/CRD.0000000000000165

[54] Lewis TC , Aberle C, Altshuler D, Piper GL, Papadopoulos J. 2019. Comparative effectiveness and safety between milrinone or dobutamine as initial inotrope therapy in cardiogenic shock. J Cardiovasc Pharm T. 24:130–138. 10.1177/1074248418797357 30175599

[55] Zhang J , et al 2020. In vivo and in vitro activation of dormant primordial follicles by EGF treatment in mouse and human. Clin Transl Med. 10:e182. 32997412 10.1002/ctm2.182 PMC7520080

[56] Pedersen T . 1970. Determination of follicle growth rate in the ovary of the immature mouse. J Reprod Fertil. 21:81–93. 5461007 10.1530/jrf.0.0210081

