# Supplementary material for: cAMP controls the balance between dormancy and activation of primordial follicles in mouse ovaries
Source: PNAS Nexus. 2023 Feb 21;2(3):pgad055. doi: 10.1093/pnasnexus/pgad055 (PMC10019762; doi:10.1093/pnasnexus/pgad055)
Supplement: pgad055_Supplementary_Data [file pgad055_supplementary_data.docx]

**Supplementary Figures**

**Fig. S1**

**
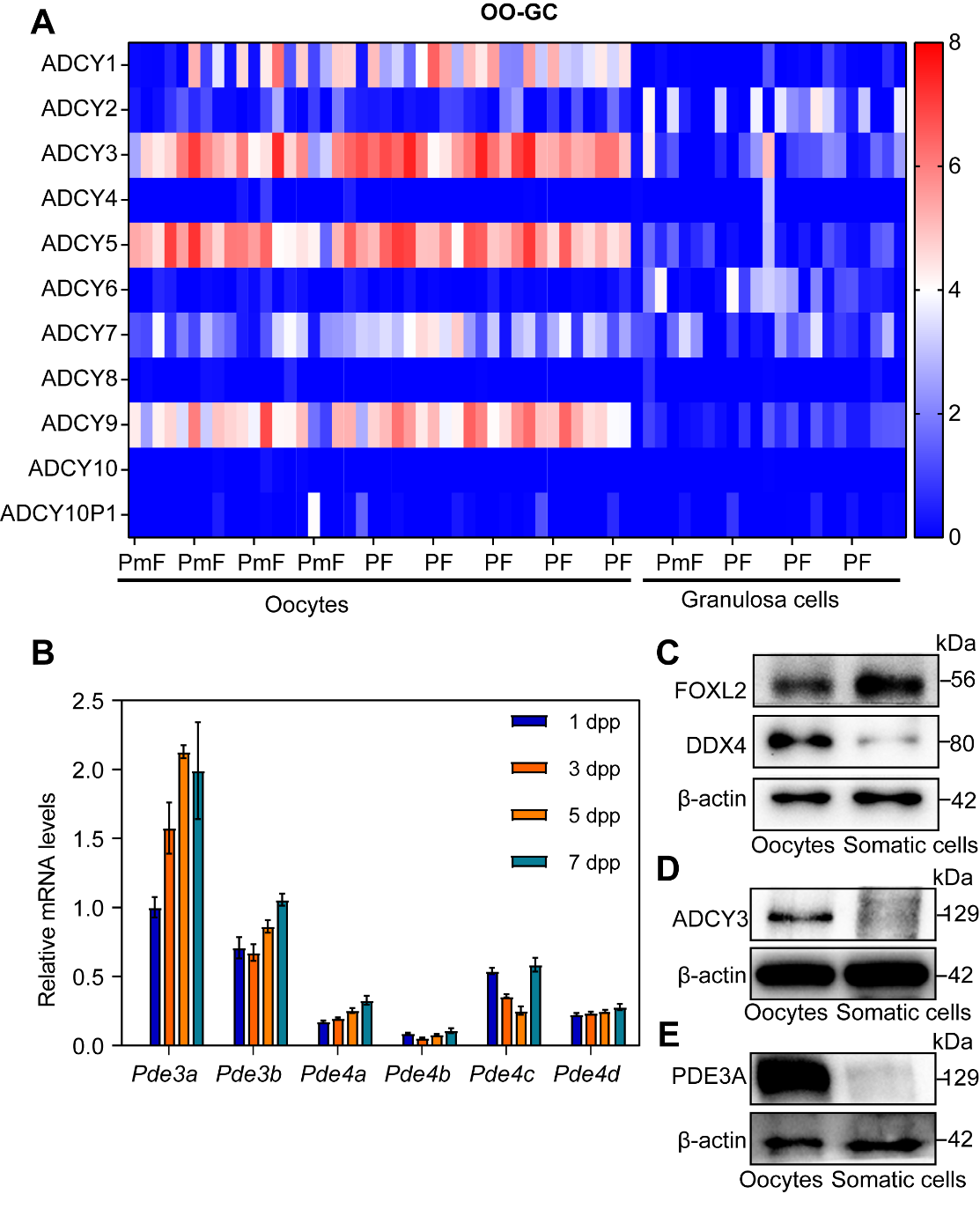
**

**Fig. S1.** ADCY3 and PDE3A were mainly expressed in the oocytes of primordial follicles and primary follicles. (A) The expression of *ADCY1-ADCY10* in human primordial follicles and primary follicles. PmF: Primordial Follicle; PF: Primary Follicle. (B) The mRNA expressions of *Pdes* in mouse ovaries were assessed by Real-time PCR. *Pde3a* was the dominant subtype during primordial follicle activation. (C) The oocytes and somatic cells were successfully isolated from 5dpp ovaries. (D) ADCY3 in oocytes was higher than that in somatic cells of 5dpp ovaries. E PDE3A was only expressed in oocytes of 5 dpp ovaries. The experiments were repeated at least three times, and representative images were shown.

**Fig. S2**

**
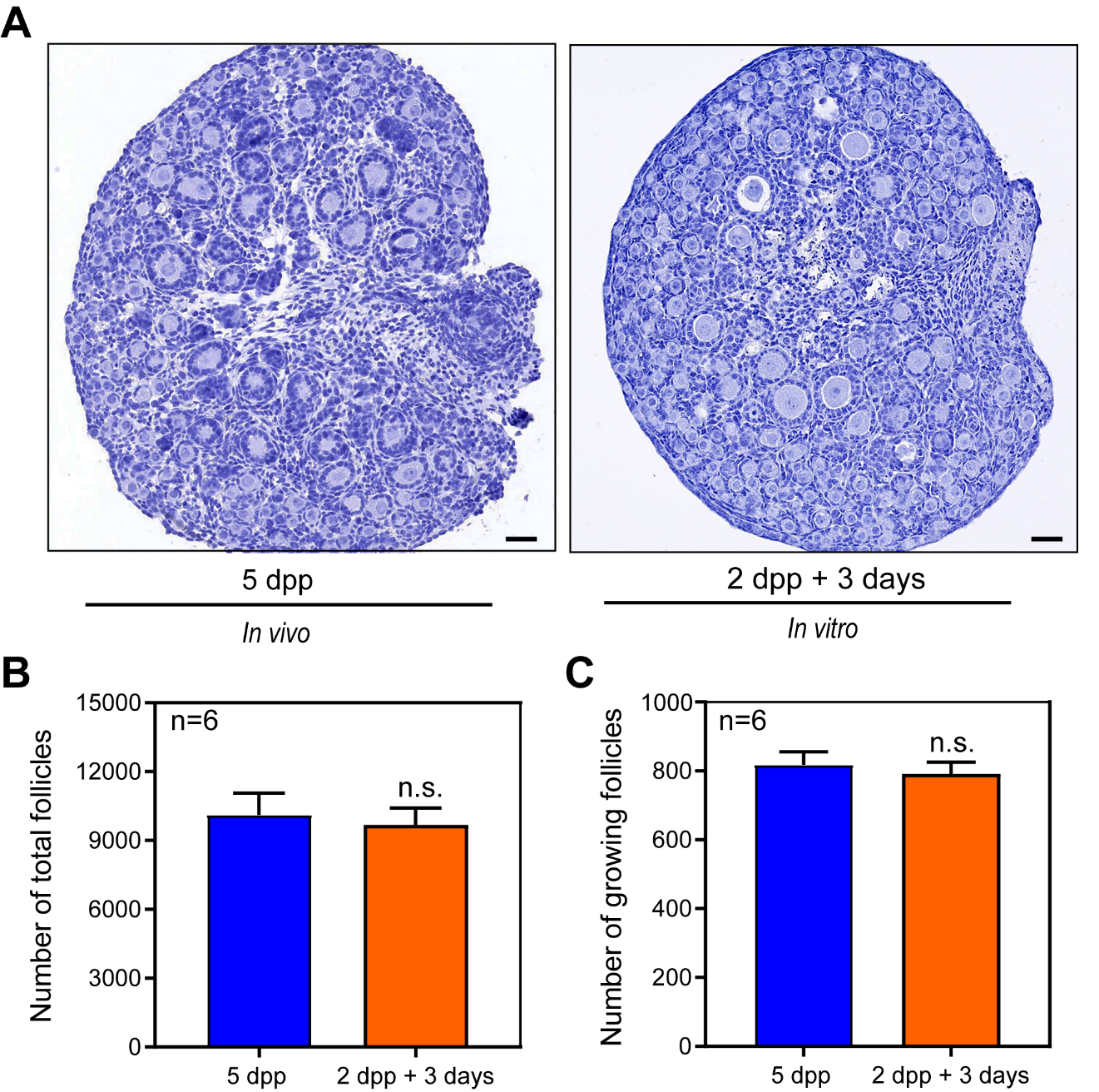
**

**Fig. S2.** Model of neonatal mouse ovaries cultured *in vitro* was established. (A) Histological analysis of ovaries at 5 dpp *in vivo* and 2 dpp cultured for 3 days *in vitro*, showed that the ovaries development normally in vitro as that *in vivo*. (B, C) The follicle counting results of total follicles and growing follicles. The experiments were repeated at least three times, and representative images were shown. Scale bars: 50 μm.

**Fig. S3**

**
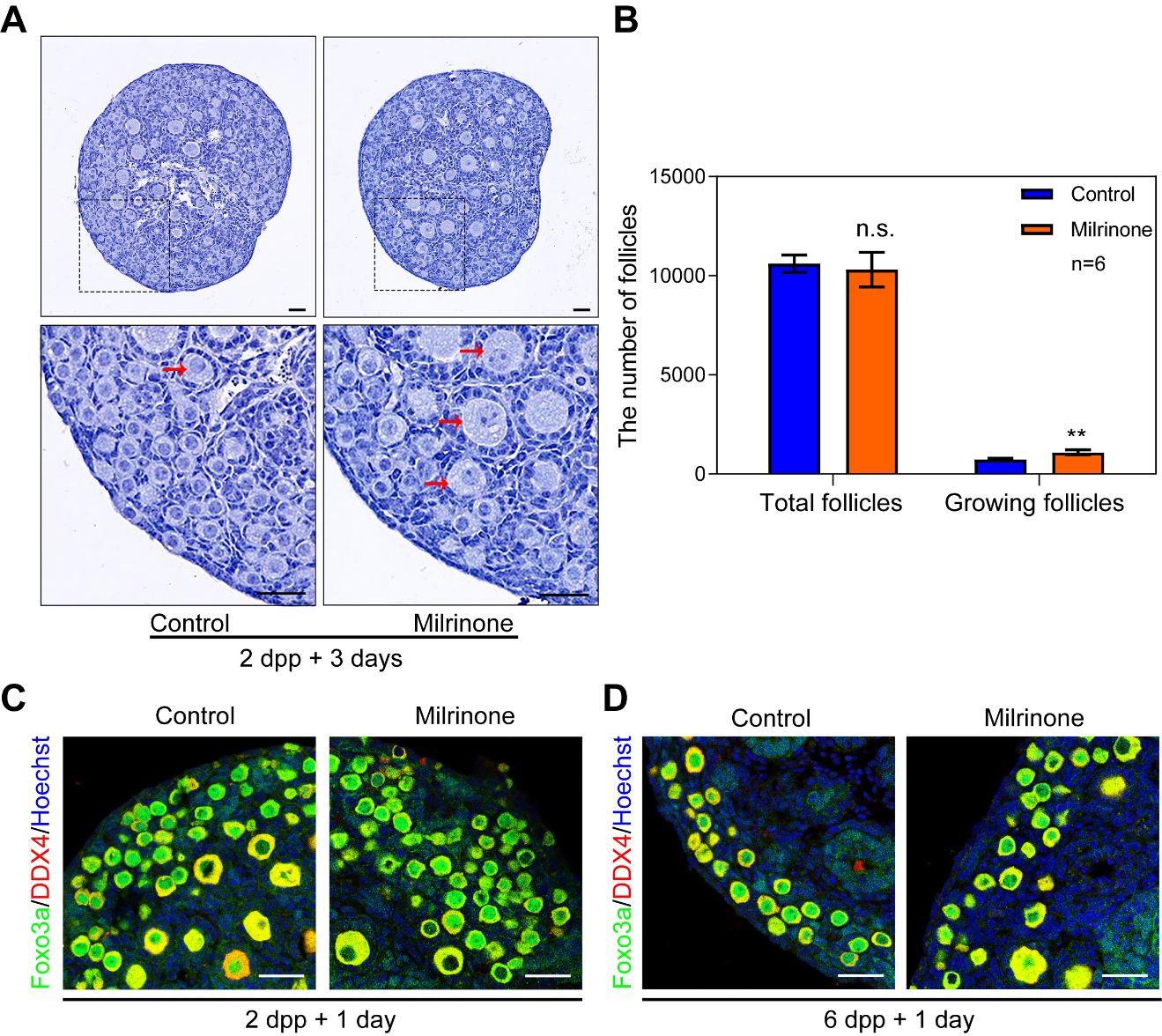
**

**Fig. S3.** Milrinone promotes the activation of primordial follicles effectually. (A, B) The activation of follicles was remarkably increased after 2 dpp ovaries with Milrinone for 3 days, while the total number of follicles was comparable in control and treated ovaries. (C, D) The proportion of CL-FOXO3a was observably increased after 2 dpp and 6 dpp ovaries were cultured with Milrinone for 1 day. The experiments were repeated at least three times, and representative images were shown. Scale bars: 50 μm.

**Fig. S4**


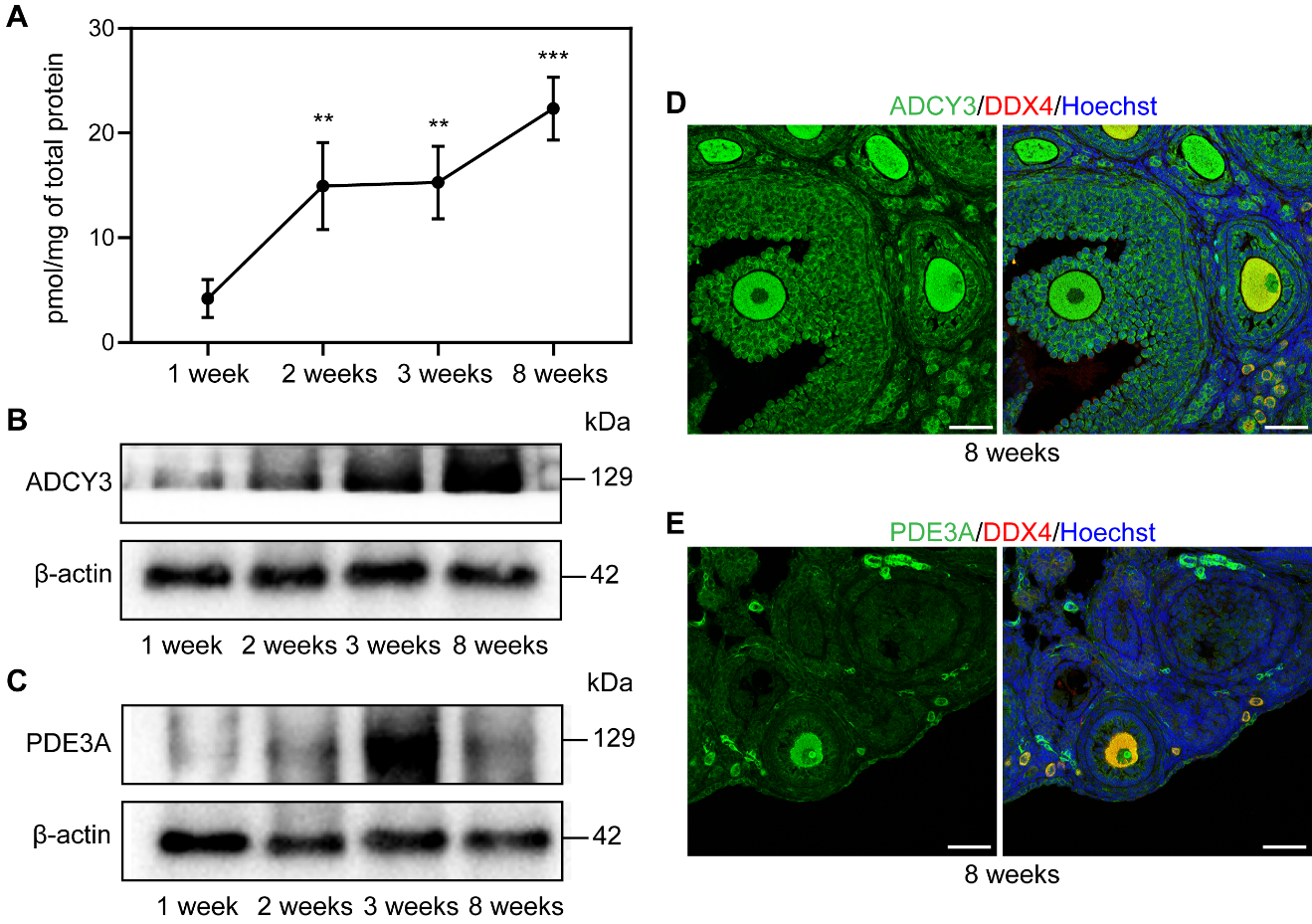


**Fig. S4.** cAMP plays a regulatory role in primordial follicle development after puberty. (A) cAMP concentration in mouse ovaries was measured from 1 week to 8 weeks by ELISA.The concentration of cAMP was significantly increased time dependently from 1 week to 2 weeks, but was kept at a constant concentration from 2 weeks to 3 weeks, and then increased to a high concentration till 8 weeks. (B) The total protein level of ADCY3 was increased from 1 week till 8 weeks. (C) The total protein level of PDE3A was increased from week 1 to 3 weeks, and decreased after puberty. (D, E) ADCY3/PDE3A were both localized in the primordial follicles and growing follicles of the mouse ovaries after puberty. (D) The localization of ADCY3 (green) in 8 weeks ovaries. The oocyte specific marker DDX4 (red). The nuclei were dyed with Hoechst (blue). (E) The localization of PDE3A (green) in 8 weeks ovaries. The oocyte specific marker DDX4 (red). The nuclei were dyed with Hoechst (blue). The experiments were repeated at least three times, and representative images were shown. Scale bars: 100 μm.

**Fig. S5**


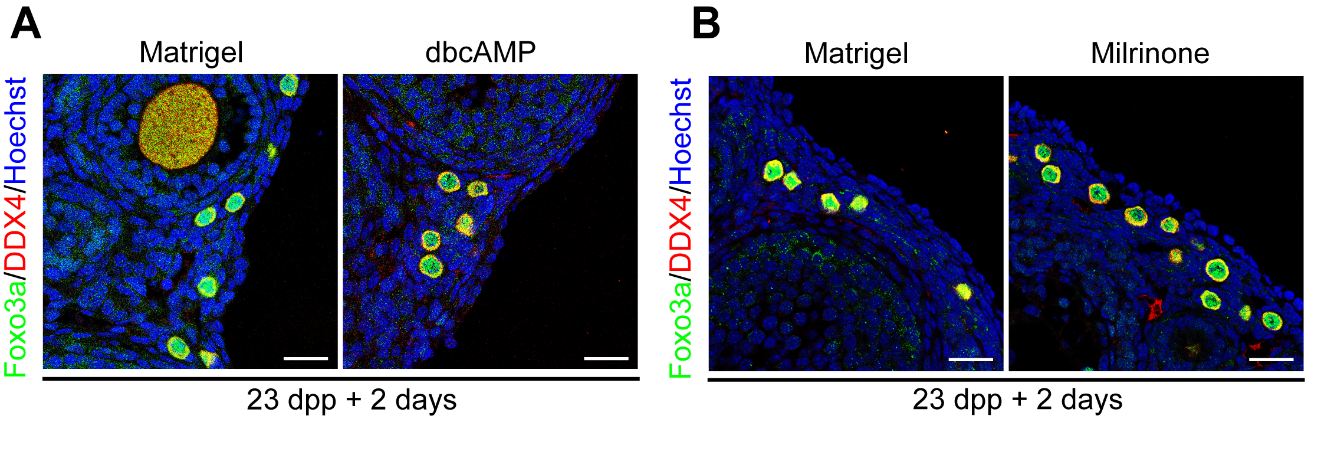


**Fig. S5.** dbcAMP and Milrinone promotes FOXO3a shuttled to the cytoplasm in oocytes after puberty. (A, B) The proportion of CL-FOXO3a was observably increased after 2 days of ovarian topical administration with dbcAMP or Milrinone. The experiments were repeated at least three times, and representative images were shown. Scale bars: 50 μm.

**Fig. S6**


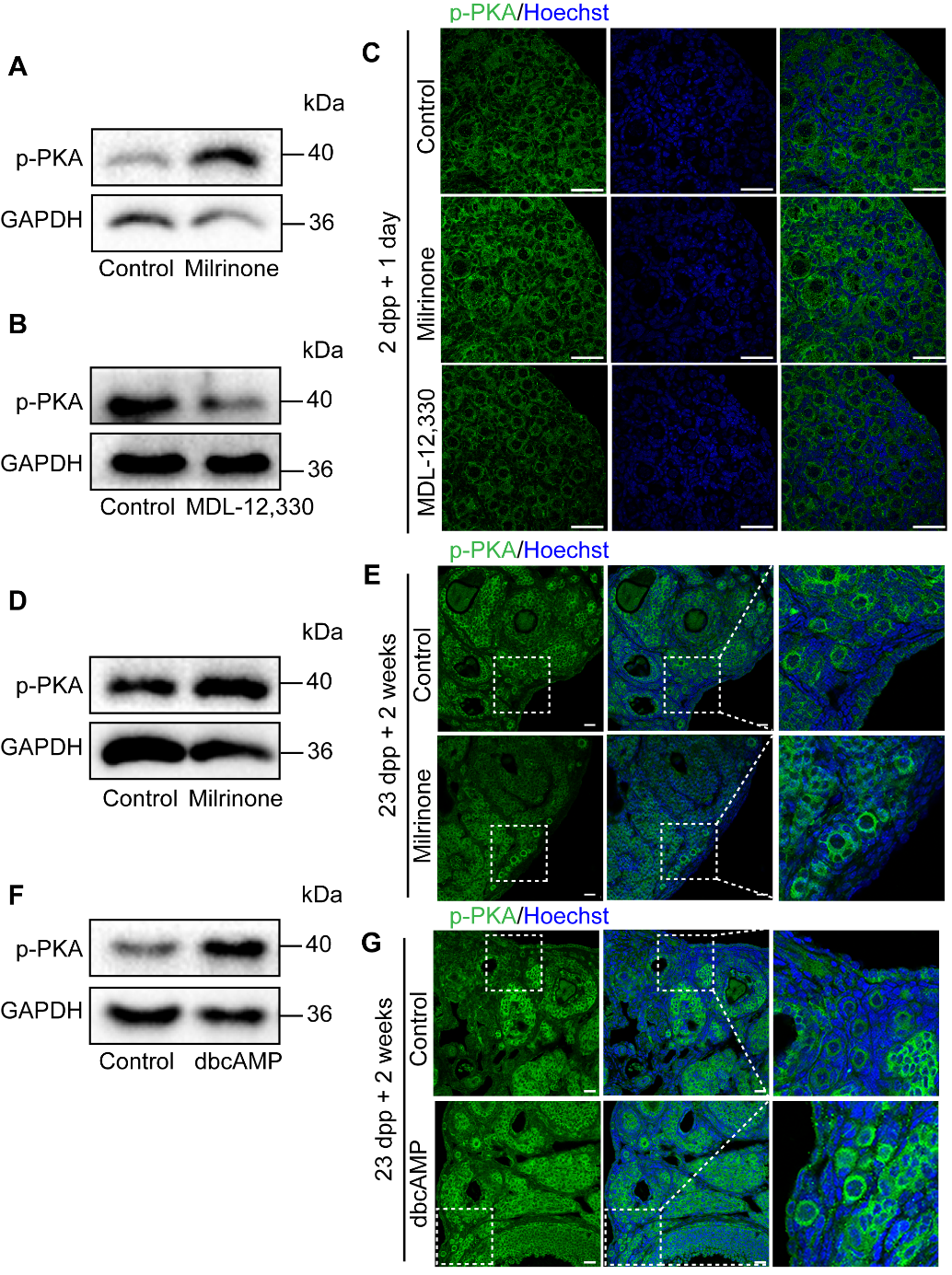


**Fig. S6.** The PKA signaling in mouse ovaries is regulated by cAMP. (A) p-PKA was increased in Milrinone group compared with controls after 2 dpp ovaries were cultured for 1 day. (B) p-PKA was decreased in MDL-12,330 group compared with controls after 2 dpp ovaries were cultured for 1 day. (C) The fluorescence intensity of ovaries cultured in each group were consistent with Western blotting results. The expression of p-PKA was increased after Milrinone treatment, while MDL-12,330 decreased the p-PKA expression level. (D-E) p-PKA was up-regulated in Milrinone group after 23 dpp ovaries were treated for 2 weeks, especially in primordial follicles. (F-G) p-PKA was up-regulated in Milrinone group after 23 dpp ovaries were treated for 2 weeks, especially in primordial follicles. The experiments were repeated at least three times, and representative images were shown. Scale bars: 50 μm.

**Fig. S7**

**
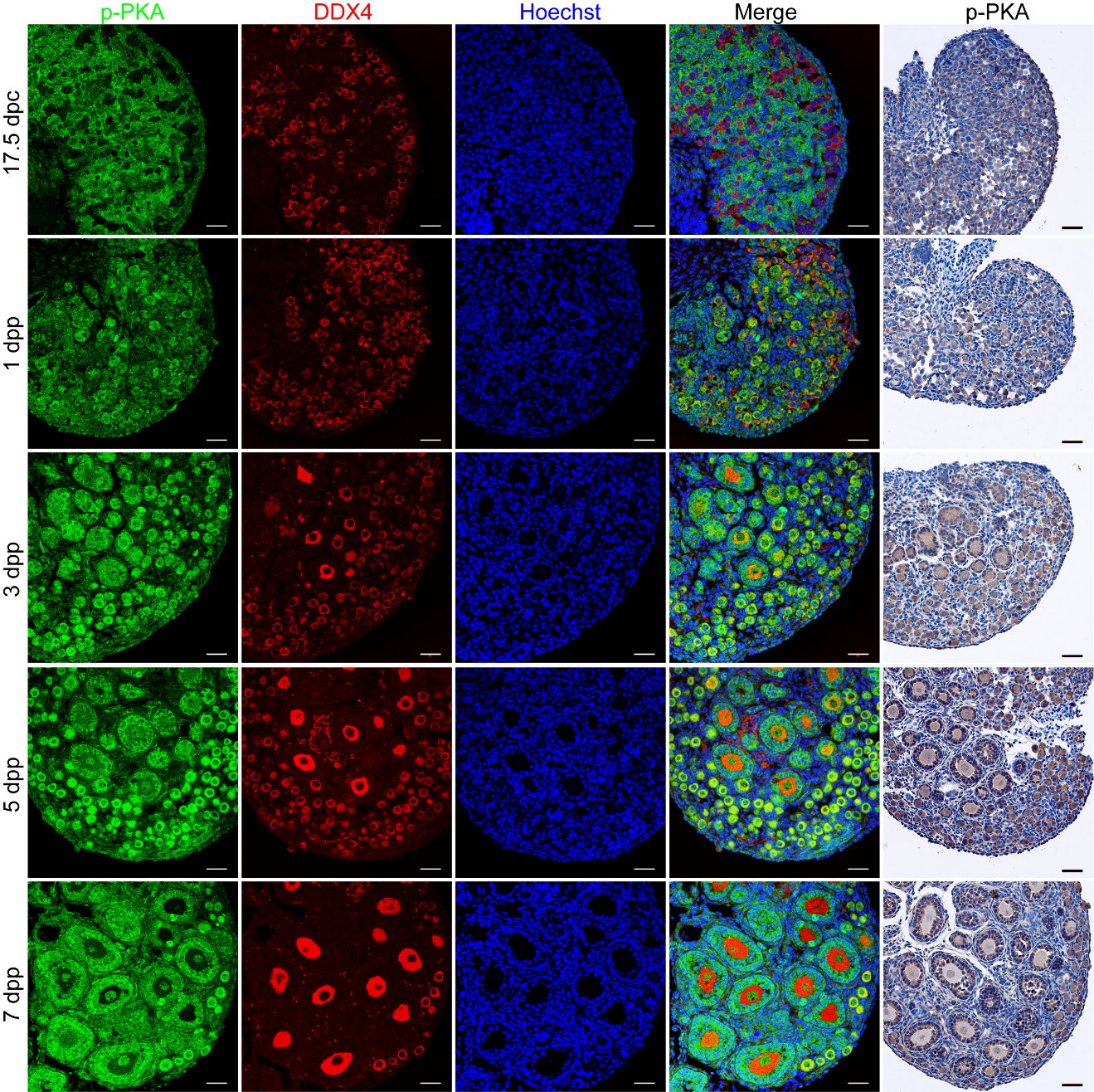
**

**Fig. S7.** The expression of p-PKA in mouse ovaries. The expression of p-PKA was very weak before the accomplishment of the primordial follicle formation. Later, p-PKA in the whole ovary of mice increased with the development of the follicles. Mouse ovaries were stained for p-PKA (green) and the oocyte marker DDX4 (red). The nuclei were dyed with Hoechst (blue). The experiments were repeated at least three times, and representative images were shown. Scale bars: 50 μm.

**Fig. S8**

**
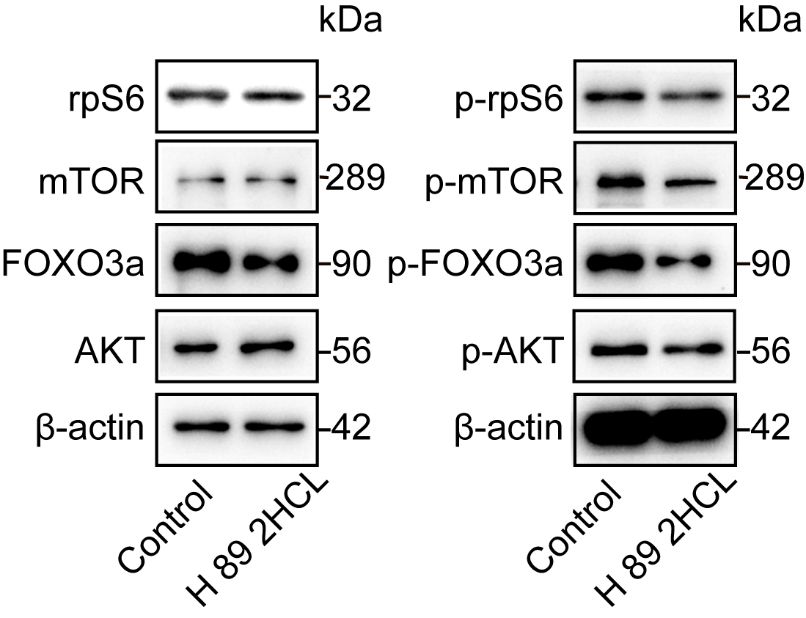
**

**Fig. S8.** Inhibition of PKA inhibited the activation of PI3K/mTOR. The PI3K and mTOR signaling pathways were activated in the ovaries treated by H 89 2HCL. The levels of total AKT, FOXO3a, mTOR and rpS6 were unchanged as compared to controls. However, the phosphorylation levels of these proteins were decreased respectively. The experiments were repeated at least three times, and representative images were shown.

**Fig. S9**

**
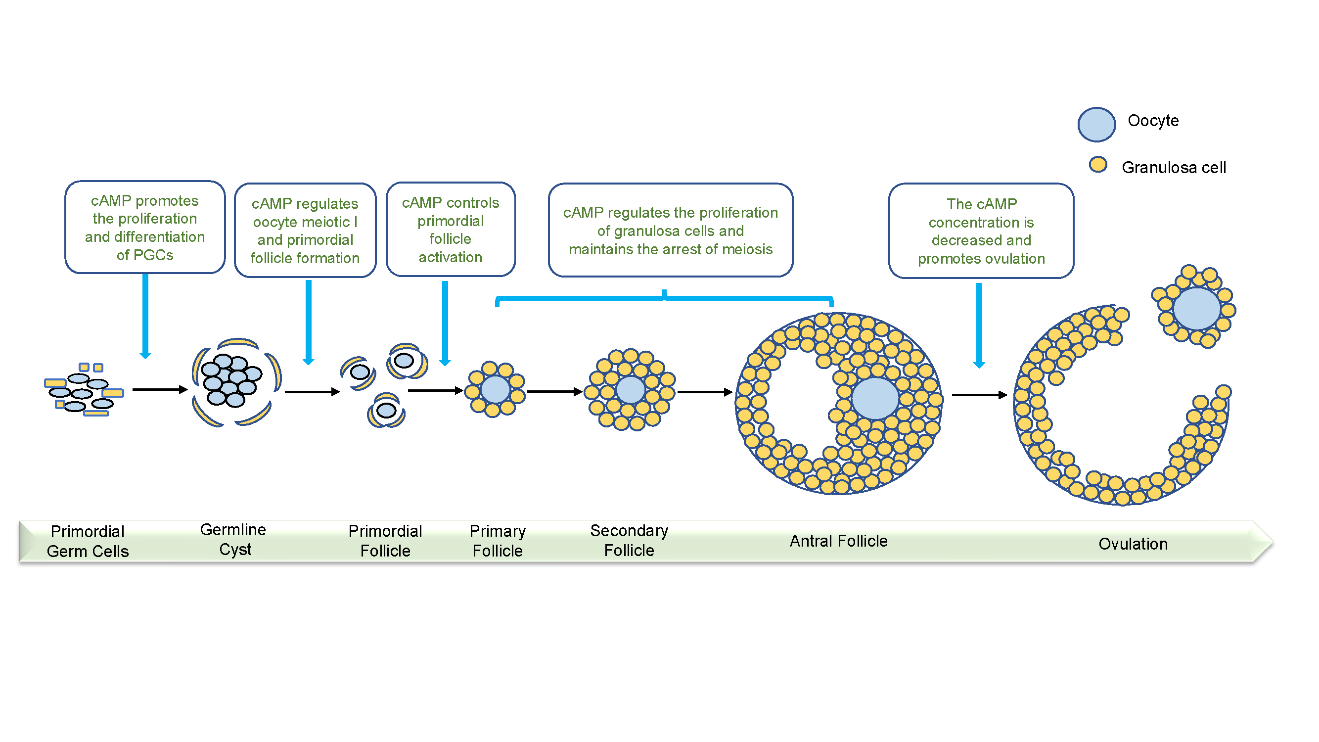
**

**Fig. S9.** The roles of cAMP in oogenesis are time dependent. cAMP is widely expressed in the cells of the mammalian ovaries. The function of cAMP in oogenesis includes regulating the proliferation and differentiation of PGCs, the proliferation of granulosa cells, and even the arrest and resumption of oocyte meiosis. PGCs: Primordial germ cells.

**Supplemental Tables**

**Table S1 Antibodies**

| **Antibodies** | **Vendors; Cat. No.** | **Source** | **Dilution/Applications** |
| --- | --- | --- | --- |
| ADCY3  PDE3A | Novus; NBP1-92683  Novus; NBP1-46181 | Rabbit  Rabbit | 1:500 (WB); 1:150 (IF)  1:500 (WB); 1:150 (IF) |
| AKT | Cell Signaling Technology; 4691 | Rabbit | 1:1000 (WB) |
| p-AKT | Cell Signaling Technology; 4060 | Rabbit | 1:1000 (WB) |
| mTOR | Cell Signaling Technology; 2983 | Rabbit | 1:1000 (WB) |
| p-mTOR | Cell Signaling Technology; 5536 | Rabbit | 1:1000 (WB) |
| DDX4 | Abcam; ab27591 | Mouse | 1:500 (WB); 1:200 (IF) |
| FOXL2 | Nouvs; NB100-1277 | Goat | 1:500 (WB) |
| FOXO3a | Cell Signaling Technology; 12829 | Rabbit | 1:1000 (WB); 1:200 (IF) |
| SYCP3 | Abcam; ab97672 | Mouse | 1:300 (IF) |
| p-FOXO3a | Abcam; ab47285 | Rabbit | 1:1000 (WB) |
| rpS6  p-rpS6  p-PKA | Cell Signaling Technology; 2217  Cell Signaling Technology; 2211  Abcam; ab75991 | Rabbit  Rabbit  Rabbit | 1:1000 (WB)  1:1000 (WB); 1:400 (IHC)  1:5000 (WB) |
| β-actin | Cwbiotech; CW0096M | Mouse | 1:5000 (WB) |
| GAPDH | Proteintech; 1049-1-AP | Rabbit | 1:2000 (WB) |
| Alexa Fluor® 488 | Thermo Scientific; A-11034 | Donkey | 1:200 (IF) |
| Alexa Fluor® 555 | Thermo Scientific; A-21127 | Donkey | 1:200 (IF) |
| Hoechst | Merck; D9542 |  | 1:500 |

**Table S2 Primers for real-time PCR**

| **Genes** | **Primers** |
| --- | --- |
| *β-actin-F*  *β-actin-R* | CATTGCTGACAGGATGCAGAAGG  TGCTGGAAGGTGGACAGTGAGG |
| *Adcy1-F* | GTGGTGGCTGCCTCGCACTT |
| *Adcy1-R*  *Adcy2-F*  *Adcy2-R*  *Adcy3-F*  *Adcy3-R*  *Adcy4-F*  *Adcy4-R*  *Adcy5-F*  *Adcy5-R*  *Adcy6-F*  *Adcy6-R*  *Adcy7-F*  *Adcy7-R*  *Adcy8-F*  *Adcy8-R*  *Adcy9-F*  *Adcy9-R*  *Adcy10-F*  *Adcy10-R*  *Pde3a-F*  *Pde3a-R*  *Pde3b-F*  *Pde3b-R*  *Pde4a-F*  *Pde4a-R*  *Pde4b-F*  *Pde4b-R*  *Pde4c-F*  *Pde4c-R*  *Pde4d-F*  *Pde4d-R* | AGCAGGGCATTGGCACCGAG  GATTGGTCTCCAGAAGTGGCAG  CCATTCAAGTGCTCCAGAGTGAC  ACACGCTCACAAACATCAACAACC  CTCCAATGACGCCAGCCAGAAC  TCCTCTTGTCTATCCTTCCTGCCTAC  ACACTCACTCCTTGGTGCCTCTT  TCGCAATGCCTACCTCAAGGAG  GCGGATTGTGTCCGATGGAGTT  TAGATCCTCGCTTCGGAGCCTA  CAACAGCAGGAAGATAGCGGCA  GACGAGATGCTGTCAGCCATTG  CACGCTCAAAGCCCTTCTCCAA  CTGCTCACAGAGACCATCTACG  CAGCAGTGATGCTTCCTTGGTC  CTTCTCTGGAGCATCTATTTCGC  CAGCTTGGTGAAAGTAAACAGGA  CTTGTGAGTGGTGTCAGGCTGA  CAAACAGCAGCTCCGTGGTGAA  ATACCTGCTCGGACTCTGAGGA  TGGCAGAGGTGGTAGTTGTCCA  GAGGTCATCGTCTGTGTCACTG  GTTAGAGAGCCAGCAGACACTG  CCGTGTTCACAGACCTGGAGAT  GGTGGTTCTCAAGCACAGACTC  ATGAGCCTCCTGGCAGACCTTA  CTGCACAGTGTACCATGTTGCG  GGAACCTCAGTACCAAGCAGAG  GCTGGTCACTTTCTTCGTCTCC  CACAGACTTGGAGATTCTCGCG  TCTAGGACCGAGGAGTCGTTGT |
